# Comparative analysis of the *MYB* gene family in seven *Ipomoea* species

**DOI:** 10.3389/fpls.2023.1155018

**Published:** 2023-03-20

**Authors:** Zengzhi Si, Lianjun Wang, Zhixin Ji, Mingming Zhao, Kai Zhang, Yake Qiao

**Affiliations:** ^1^ Hebei Key Laboratory of Crop Stress Biology, Hebei Normal University of Science and Technology, Qinghuangdao, Hebei, China; ^2^ Institute of Food Corps, Hubei Academy of Agricultural Sciences, Wuhan, Hubei, China

**Keywords:** *Ipomoea* species, MYB gene family, phylogenetic analysis, duplication analysis, gene expression, stresses response, sweet potato

## Abstract

The MYB transcription factors regulate plant growth, development, and defense responses. However, information about the *MYB* gene family in *Ipomoea* species is rare. Herein, we performed a comprehensive genome-wide comparative analysis of this gene family among seven *Ipomoea* species, sweet potato (*I. batatas*), *I. trifida*, *I. triloba*, *I. nil*, *I. purpurea*, *I. cairica*, and *I. aquatic*, and identified 296, 430, 411, 291, 226, 281, and 277 *MYB* genes, respectively. The identified *MYB* genes were classified into five types: *1R*-*MYB* (*MYB*-related), *2R*-*MYB* (*R2R3*-*MYB*), *3R*-*MYB* (*R1R2R3*-*MYB*), *4R*-*MYB*, and *5R-MYB*, and the *MYB-related* or *R2R3*-*MYB* type was the most abundant *MYB* genes in the seven species. The *Ipomoea MYB* genes were classed into distinct subgroups based on the phylogenetic topology and the classification of the *MYB* superfamily in Arabidopsis. Analysis of gene structure and protein motifs revealed that members within the same phylogenetic group presented similar exon/intron and motif organization. The identified *MYB* genes were unevenly mapped on the chromosomes of each *Ipomoea* species. Duplication analysis indicated that segmental and tandem duplications contribute to expanding the *Ipomoea MYB* genes. Non-synonymous substitution (Ka) to synonymous substitution (Ks) [Ka/Ks] analysis showed that the duplicated *Ipomoea MYB* genes are mainly under purifying selection. Numerous *cis*-regulatory elements related to stress responses were detected in the *MYB* promoters. Six sweet potato transcriptome datasets referring to abiotic and biotic stresses were analyzed, and *MYB* different expression genes’ (DEGs’) responses to stress treatments were detected. Moreover, 10 sweet potato *MYB* DEGs were selected for qRT-PCR analysis. The results revealed that four responded to biotic stress (stem nematodes and *Ceratocystis fimbriata* pathogen infection) and six responded to the biotic stress (cold, drought, and salt). The results may provide new insights into the evolution of *MYB* genes in the *Ipomoea* genome and contribute to the future molecular breeding of sweet potatoes.

## 1 Introduction

The MYB transcription factor (TF) family, as one of the most prominent TF families in the plant, is composed of three conservative functional domains: DNA-binding domain (DBD), transcriptional activation domain (TAD), and incompletely defied negative regulatory region (NRD). Among the three, DBD is the most conservative and is generally known as the MYB domain (Frampton, 2004). The MYB domain consists of 51–52 conserved amino acids and spacing coding sequences, and every 18 amino acids has a regular spacing of tryptophan residues. This amino acid residue expands the MYB domain into a helix (HTH) structure (Frampton, 2004). According to the total number of adjacent MYB repetitions, MYB TF can be divided into four categories, namely, 1R-MYB (MYB-related), 2R-MYB, 3R-MYB, and 4R-MYB, which contain the corresponding number of MYB repeats, respectively (Dubos et al., 2010).

1R-MYB, also called MYB-related, generally but not always include a single or partial MYB iteration (Klempnauer et al., 1982). Based on the highly conserved motif, the MYB-related TFs can be divided into subgroups (Liu et al., 2020b; Arce-Rodríguez et al., 2021; Yan et al., 2022). The MYB-related TFs play essential roles in plant seed germination and root growth (Zhao et al., 2019), leaf senescence and epidermal cell patterning (Lee and Schiefelbein, 1999; Machado et al., 2009; Park et al., 2018), biosynthesis (Sinaga et al., 2021), the regulation of circadian rhythms (Alabadí et al., 2001; Mizoguchi et al., 2002; Okada et al., 2009; Kamioka et al., 2016), and stress responses (Dubos et al., 2010; Park et al., 2018; Zhao et al., 2019; Hoseinpour et al., 2021).

The *2R-MYB* genes, named R2R3-MYB, were usually the largest *MYB* gene subfamily in green plants (Martin and Paz-Ares, 1997). They play essential roles in plant lifetime, including (1) primary and secondary metabolites, such as phenylpropanoid metabolism (Liu et al., 2015; Tang et al., 2021), flavonoid biosynthesis (Czemmel et al., 2012; Lin et al., 2021; Zhao et al., 2022b), anthocyanin biosynthesis (Naing and Kim, 2018; Upadhyaya et al., 2021; Wang et al., 2022c), chlorogenic acid biosynthesis (Tang et al., 2021), indolic and aliphatic glucosinolate biosynthesis (Gigolashvili et al., 2009), and apigenin biosynthesis (Wang et al., 2022b); (2) cell fate and identity, for instance, epidermis and root formation (Du et al., 2009); (3) developmental processes, like embryogenesis (Wang et al., 2009), pollen development (Liu et al., 2021), male sterility (Yu et al., 2021), and plant trichome development (Shangguan et al., 2021); and (4) responses to biotic and abiotic stresses, including diseases (Shen et al., 2017; Gu et al., 2020; Hawku et al., 2022), high salinity (Wang et al., 2021a; Du et al., 2022), cold (Dong et al., 2021), heat (Wu et al., 2021b), and drought (Lv et al., 2021; Wu et al., 2021a).

The 3R-MYB TFs in plants recognize mitosis-specific activator (MSA) elements (Ito et al., 1998; Ma et al., 2009) and play an essential role in cell-cycle regulation. The plant 3R-MYB TFs regulate the G2/M transition (Ito et al., 2001). The recognized DNA element (MSA) was in the upstream promoter region of G2/M-phase-specific genes, which is both necessary and sufficient for driving G2/M-phase-specific gene expression (Ito et al., 2001; Kato et al., 2009). The plant 3R-MYB TFs were often classed into three subgroups: A, B, and C (Ito, 2005). The 3R-MYB TFs in the A and B subgroups were reported to be involved in cell-cycle regulation (Ito et al., 2001; Kato et al., 2009; Haga et al., 2011), and the ones in the C group participated in both cell-cycle and abiotic stresses (Dai et al., 2007; Ma et al., 2009). It was also found that 3R-MYB inhibited plant trichome development and inhibited flavonoid biosynthetically (Dubos et al., 2008; Matsui et al., 2008). The 3R-MYB gene, *PhMYBx* in petunia, downregulated anthocyanin synthesis (Koes et al., 2005; Chezem and Clay, 2016). The 4R-MYB TFs, representing the smallest subfamily of MYB TFs, contain four R1/R2-like repeats. Little is known about their functions in plants. Recently, the 4R-MYB protein SNAPc4 in Arabidopsis was reported as part of the SNAP complex involved in snRNA gene transcription, and *AtSNAPc4* is proved to be an essential gene in gametophyte and zygote development (Thiedig et al., 2021).

Since the first plant *MYB* gene (*ZmMYBC1*) was cloned and reported to be involved in pigment biosynthesis in *Zea maize* (Paz-Ares et al., 1987), the characterization and analysis of *MYB* genes have been conducted in different plant species. In most cases, the *2R-MYB* genes were the most abundant, followed by *MYB-related* genes and *3R-MYB* genes, and the *4R-MYB* gene group was the smallest (Dubos et al., 2010). However, the number of *MYB* genes varied widely among species, ranging from less than 100 to more than 500. For instance, 198 *MYB* genes in Arabidopsis (Chen et al., 2006), 183 in *Oryza sativa* (Chen et al., 2006), 475 in *Brassica rapa* ssp. *Pekinensis* (Saha et al., 2016), 524 in *Gossypium hirsutum* (Salih et al., 2016), 174 in *Morella rubra* (Cao et al., 2021), 217 in *Solanum tuberosum* (Li et al., 2021c), 182 in *Casuarina equisetifolia* (Wang et al., 2021b), and 54 in *Mangifera indica* (Zhang et al., 2022) were identified and analyzed. The number of *MYB* genes greatly varied in the species of the same genus. For example, in the *Solanum* genus, 127 *MYB* genes were reported in *S. lycopersicum* (Li et al., 2016) whereas 217 in *S. tuberosum* (Li et al., 2021c); in the *Musa* genus, 305 *MYB* genes were identified in *M. acuminate* whereas 251 in *M. balbisiana* (Tan et al., 2020). To date, information about the comparative analysis of *MYB* genes in closely related species (the same genus) is still limited.

The genus *Ipomoea* (family Convolvulaceae) contains 600–700 species (Nimmakayala et al., 2011), many of which are of considerable importance either as medicinal plants or as ornamental plants (Nimmakayala et al., 2011). The sweet potato (*I. batatas*), serving as the seventh most important crop in the world, is an essential source of calories, proteins, vitamins, and minerals for humanity (Bovell-Benjamin, 2007; Yang et al., 2017). *I. trifida* and *I. triloba*, two diploid relatives of sweet potatoes, can be used as model plants to facilitate sweet potato breeding (Wu et al., 2018). *I. nil* and *I. purpurea* are ideal plants for researching photoperiodic flowering and flower coloration (Hoshino et al., 2016). *I. cairica* (L.) is a perennial vine plant that blooms all year round and has been widely introduced to subtropical, subtropical, and temperate regions as ornamental plants for the landscape. It also has the characteristics of medicinal value because it contains a large number of bioactive compounds. In many countries, a decoction of the whole plant is used to treat tuberculosis, cough, asthma, liver cirrhosis, and jaundice (Jiang et al., 2022). Water spinach (*I. aquatica*) is one of Asia’s most popular green leafy vegetables, with both aquatic and terrestrial characteristics (Hao et al., 2021).

Related information is limited compared to the importance of the *MYB* gene family and *Ipomoea* species, particularly for sweet potatoes. For the sweet potato, the researchers mainly focused on researching a few *2R-MYB* genes in the past years. *IbMYB1* increases the anthocyanin content and the resistance ability of potatoes (Cheng et al., 2013); the *IbMYB1a* gene induces anthocyanin accumulation in Arabidopsis (Chu et al., 2013); and overexpression of the *IbMYB1* gene in an orange-fleshed sweet potato cultivar produces a dual-pigmented transgenic sweet potato with improved antioxidant activity (Park et al., 2015). *IbMYB116* was reported to enhance the drought tolerance of Arabidopsis (Zhou et al., 2019). A single amino acid mutant in the EAR motif of *IbMYB44.2* could reduce the inhibition of anthocyanin accumulation in the purple-fleshed sweet potato (Li et al., 2021a). *IbMYB48* (a sweet potato R2R3-MYB gene) confers enhanced tolerance to salt and drought stresses in transgenic Arabidopsis (Zhao et al., 2022a). *IbMYB308* improves salt stress tolerance in transgenic tobacco (Wang et al., 2022a). However, there is no comparative analysis of the *MYB* gene family of *Ipomoea* species, and knowledge of *MYB* genes of this genus is limited.

This study conducted a genome-wide comparative analysis of the *MYB* gene family in seven *Ipomoea* species. There were 296, 430, 411, 291, 226, 281, and 277 *MYB* genes identified from sweet potato (*I. batatas*), *I. trifida*, *I. triloba*, *I. nil*, *I. purpurea*, *I. cairica*, and *I. aquatic*, respectively. The identified *MYB* genes were then subjected to phylogenetic analysis, gene structure investigation, chromosome location, syntenic analysis, non-synonymous substitution (Ka) to synonymous substitution (Ks) [Ka/Ks] calculation, *cis*-regulatory element (CRE) detection, and expression profile analysis. Then, 10 sweet potato *MYB* different expression genes (DEGs) were selected for qRT-PCR analysis. The results showed that four responded to biotic stress (stem nematodes and *Ceratocystis fimbriata* pathogen infection) and six responded to abiotic stress (cold, drought, and salt). These results are likely to give a new view on the evolution of the *MYB* gene in *Ipomoea* species. They are conducive to the development of molecular breeding in sweet potatoes in the future.

## 2 Materials and methods

### 2.1 Data resources

The whole genome sequences and annotated files of the seven *Ipomoea* species were obtained from the following open-access databases: sweet potato from the *I. batatas* Genome Browser (http://public-genomes-ngs.molgen.mpg.de/SweetPotato/), *I. trifida* and *I. triloba* from GenBank BioProject (accessions numbers PRJNA428214 and PRJNA428241), *I. nil* from the *I. nil* web (http://viewer.shigen.info/asagao/index.php), *I. purpurea* from the CoGe platform (https://genomevolution.org/coge/GenomeInfo.pl?gid=58735), *I. cairica* from AGIS (ftp://ftp.agis.org.cn/~fanwei/Ipomoea_cairica_genome_v1), and *I. aquatic* from BIGD (PRJCA002216). The MYB protein sequences of Arabidopsis were downloaded from the TAIR database (https://www.arabidopsis.org) (Chen et al., 2006).

### 2.2 Identification of *MYB* genes in *Ipomoea* species

To identify the *MYB* genes in *Ipomoea* species, two strategies were adopted. First, the protein sequences of the seven *Ipomoea* species were searched for the MYB domain (Pfam accession number: PF00249) using HMMsearch (ver. 3.1b2) with default parameters. Second, the MYB protein sequences of Arabidopsis were used as queries to a search against the protein database of each *Ipomoea* species by BLASTP (ver. 2.10.0+) with an E-value cutoff of 1e-10 (Altschul et al., 1990). The protein sequences obtained from the two strategies were merged, and the redundant ones were removed. Then, all of the MYB proteins were subjected to a search against the Pfam database (release 35; adopted Pfam A) using PfamScan.pl (v1.6) with default parameters, search against the Conserved Domain Database (CDD) database (ver. 3.20) employing Reverse Position-Specific BLAST (RPS–BLAST) (ver. 2.10.0+) with an e-value cutoff of 1e-10, and search against the Simple Modular Architecture Research Tool (SMART) database (http://smart.embl-heidelberg.de/) by performing SMART_batch.pl (http://smart.embl-heidelberg.de/help/SMART_batch.pl) with default parameters. The proteins confirmed by all three databases were considered candidate MYB TFs.

### 2.3 Molecular weight, isoelectric point, and subcellular localization analysis of *Ipomoea* MYB proteins

The molecular weight (MW) and isoelectric point (pI) for each MYB protein were analyzed by using ExPASy (http://www.expasy.ch/tools/pi_tool.html). The online website WoLF PSORT (https://wolfpsort.hgc.jp/) was employed for predicting the subcellular localization of the *Ipomoea* proteins.

### 2.4 Identification of conserved motifs of the *Ipomoea MYB* genes

The MYB protein sequence motif analysis was performed using the online MEME Suite (https://meme-suite.org/meme/) (Bailey et al., 2009). The maximum detecting number of motifs was designed to identify 20 motifs and the site distribution was set as any, whereas other parameters were set as default.

### 2.5 Sequence alignment and phylogenetic analysis of *Ipomoea* MYB proteins

The *Ipomoea* and Arabidopsis MYB protein sequences were aligned using Clustal Omega (Sievers et al., 2011; Sievers and Higgins, 2018). The obtained aligned sequences were submitted to IQ-TREE for phylogenetic analysis using the maximum likelihood approach (Nguyen et al., 2015). The branch support values were calculated by SH-aLRT and UFBoot2 with 1,000 bootstrap replicates (Anisimova et al., 2011).

### 2.6 Protein motif compositions and gene structures of *Ipomoea MYB* genes

Based on the results of the online MEME Suite, phylogenetic analysis, and the gff3 files of the genomes, the *Ipomoea MYB* genes identified above were submitted to TBtools for protein motif compositions and gene structure analysis and picture drawing (Chen et al., 2020).

### 2.7 Chromosome distribution and duplication pattern analysis of the *Ipomoea MYB* genes

The *MYB* genes with chromosomal positions were mapped on the chromosomes of seven *Ipomoea* species with MapChart (ver. 2.30) (Voorrips, 2002). The potential duplicated *MYB* genes in the *Ipomoea* genome were analyzed with MCScanX software (Wang et al., 2012). During this stage, the protein sequences of *Ipomoea* species were compared against themselves by running the BLASTP (ver. 2.10.0+) program with an E-value of 1e–10 (Altschul et al., 1990). The final output of the duplicated analysis was visualized with the CIRCOS software (ver. 0.66) (Krzywinski et al., 2009).

### 2.8 Syntenic analysis of *MYB* genes in the seven *Ipomoea* genomes

Syntenic block in the genomes of the seven *Ipomoea* species was analyzed using MCScan software (Python version3) (Tang et al., 2008) with the default parameters (VanBuren et al., 2018; Li et al., 2021b). The gene models were aligned with LAST, and hits were filtered to locate the best 1:1 syntenic blocks (pairs) and were visualized in the dot-plot script using the JCVI package (Tang et al., 2008).

### 2.9 Ka/Ks analysis of duplicated and syntenic *MYB* genes

Both duplicated and syntenic *MYB* gene pairs of the *Ipomoea* species were selected for the non-synonymous substitution (Ka) to synonymous substitution (Ks) [Ka/Ks] calculation with TBtools (Chen et al., 2020).

### 2.10 Promoter analysis of *Ipomoea MYB* genes

The 1.5-kb promoter sequences of the *Ipomoea MYB* genes were submitted into PLANT CARE (http://bioinformatics.psb.ugent.be/webtools/plantcare/html/, accessed on 21 March 2021) for identification of the putative *cis*-elements (Magali et al., 2002).

### 2.11 Expression profile of *MYB Ipomoea* genes

Six transcriptome bio project datasets referring to abiotic and biotic stresses were selected for expression profile analysis of the sweet potato *MYB* genes. Three bio project datasets (PRJNA341328 for cold, PRJNA413661 for drought, and PRJNA631585 for salt) referring to abiotic stress and one bio project (PRJNA429283 for root-knot nematode) referring to biotic stress were obtained from the NCBI database. The other two were our in-house transcriptome datasets (unpublished) for sweet potato stem nematodes and *C. fimbriata* resistance of four sweet potato cultivars or lines (sweet potato stem nematode-susceptible cultivar, “Tengfei,” sweet potato stem nematode-resistant line, “JK20,” *C. fimbriata*-susceptible cultivar, “Santiandao,” and *C. fimbriata*-resistant line, “JK274”). In each comparing case, reads were treated as DEGs if |log2FC|>1 and FDR ≤ 5%. Thus, the mean log2FC value for each DEG was calculated. The heat map was constructed to visualize the distribution of the expression level of genes using the fragments per kilobase per million (FPKM) value in MeV software (Howe et al., 2011). The gene FPKM data of *I. trifida* and *I. triloba* were downloaded from the sweet potato database (http://sweetpotato.plantbiology.msu.edu/).

### 2.12 RNA isolation and quantitative qRT-PCR analysis

Xu32 (susceptible cultivar) and JK328 (resistant line) were selected for cold, salt, and drought stress treatments. The cuttings about 25 cm in length from 6-week-olds of them grown in a field were cultured in the Hoagland solution for 3 days to survive: for cold stress treatment, the cuttings were then placed at 28°C (control) and 16°C (cold stress), respectively; for salt stress treatment, the cuttings were cultured in the Hoagland solution with 0 and 86 mM NaCl, respectively; and for drought stress treatments, the cuttings were cultured in Hoagland solution with 0% and 30% PEG 6000. Samples were collected at seven time points (0, 2, 4, 6, 12, 24, and 48 h) after the treatments. The cultivars Tengfei (susceptible cultivar) and JK20 (resistant line) were inoculated with sweet potato stem nematodes (Gao et al., 2011), and the cultivars Santiandao (susceptible cultivar) and JK274 (resistant line) were inoculated with *C. fimbriata* (Muramoto et al., 2012). Samples were collected at seven time points (0 h, 6 h, 12 h, 1 day, 2 days, 4 days, and 6 days) after the injection. Root samples without injection were used as a control. Then, the total RNA of the samples was isolated using RNAprep Pure Plant Kit (Tiangen Biotech, Beijing, China) and first-strand cDNA was synthesized by QuantScript Reverse Transcriptase Kit (Tiangen Biotech, Beijing, China). The sweet potato β-actin gene (GenBank AY905538) was used as a control and to normalize the relative quantities of the three individual targeted DEGs based on its consistency across the different time points of each treatment. Three biological replicates were performed at each time point, and the gene expression changes were calculated using the 2^–ΔΔCt^ method for each sample (Schmittgen and Livak, 2008). Using the primers (**Table S1**) generated with Primer-BLAST software (Ye et al., 2006), qRT-PCR was performed as described previously (Zhai et al., 2016).

## 3 Results

### 3.1 Identification of the *MYB* genes in the seven *Ipomoea* species

A total of 2,212 *MYB* genes were identified from the seven *Ipomoea* species: 296 from sweet potato (*I. batatas*), 430 from *I. trifida*, 411 from *I. triloba*, 291 from *I. nil*, 226 from *I. purpurea*, 281 from *I. cairica*, and 277 from *I. aquatic* (**Tables S2**, **S3**). The number of MYB TFs in the seven investigated *Ipomoea* species varied. According to the total number of adjacent MYB repetitions, the identified MYB TFs were classed into five types: MYB-related (1R-MYB), R2R3-MYB (2R-MYB), 3R-MYB, 4R-MYB, and 5R-MYB (**Tables S2**, **S3**). Among these types, the MYB-related or R2R3-MYB was the most abundant in the seven *Ipomoea* species. At the same time, the percentages of 3R-MYB, 4R-MYB, and 5R-MYB were comparatively small and no 4R-MYB TFs were detected in *I. nil*, *I. purpurea*, *I. cairica*, and *I. aquatic* (**Tables S2**, **S3**). In the sweet potato (*I. batatas*), *I. nil*, *I. cairica*, and *I. aquatic*, the number of R2R3-MYB TFs was more than the MYB-related ones, whereas in *I. trifida*, *I. triloba*, and *I. purpurea*, the MYB-related TFs were more dominant than the R2R3-MYB ones, especially in *I. purpurea*; the proportion of MYB-related TFs (67.26%) was equivalent to 2.17 times of that of R2R3-MYB ones (**Tables S2**, **S3**).

### 3.2 Molecular weight, isoelectric point, and subcellular localization analysis of *Ipomoea* MYB proteins

The average length of the *Ipomoea* MYB TFs was 377.43 amino acids, with an average exon number of 4.41(**Tables S2**, **S3**). The average of the calculated molecular weight (MW) of the *Ipomoea* MYB TFs was 41.96 kDa, ranging from 8.66 to 219.26 kDa. In all of the seven *Ipomoea* species, the average length, the average exon number, and the average MW of the *MYB* genes were increased with the increase in the number of adjacent MYB repetitions, except for that of the *R2R3-MYB* genes which was smaller than that of *MYB-related* genes (**Tables S2**, **S3**). The predicted isoelectric point (pI) of the *Ipomoea* MYB TFs ranged from 4.15 to 11.23, with an average pI of 7.15. Moreover, the average pI of 4R-MYB and 5R-MYB TFs was larger than that of other types of MYB TFs in the seven *Ipomoea* species (**Tables S2**, **S3**). The grand average of hydropathicity of the *Ipomoea* MYB TFs was -0.70, ranging from -1.53 to 0.18 (**Tables S2**, **S3**). Subcellular localization analysis showed that the majority of the *Ipomoea* MYB TFs (>90%) were predicted to be localized in the nucleus, with a small set of them predicted to localize in other subcellular locations, such as chloroplast, cytosol, and mitochondrial (**Tables S4**, **S5**).

### 3.3 Conserved motif analysis of the *Ipomoea* MYB proteins

**Figure 1:**
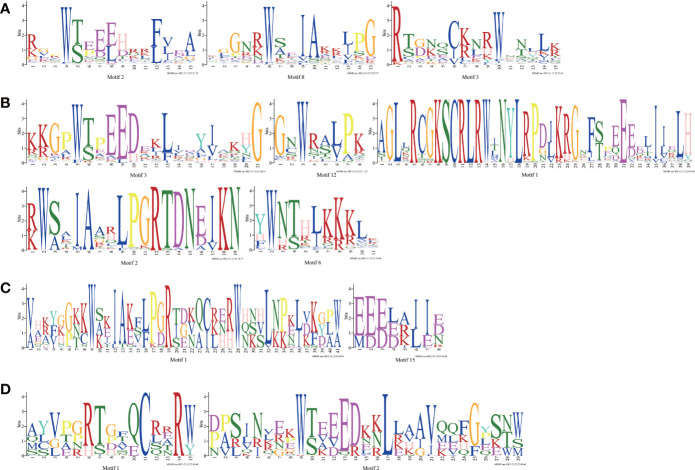
MYB repeats of the proteins in *Ipomoea* MYB TFs. **(A)** MYB repeats of *Ipomoea* MYB-related TFs; **(B)** MYB repeats of *Ipomoea* R2R3-MYB TFs; **(C)** MYB repeats of *Ipomoea* 3R-MYB TFs; **(D)** MYB repeats of *Ipomoea* 4R- and 5R-MYB TFs.

To understand the conserved domains of the *Ipomoea* MYB proteins, the Motif Elicitation (MEME) analysis was performed on each type of MYB TFs identified above. Twenty conserved motifs were found in the *Ipomoea* MYB-related TFs, R2R3-MYB TFs, 3R-MYB TFs, and the other (4R- and 5R-MYB) TFs, respectively (**Figure S1**). As shown in **Figure 1**, in addition to the number of adjacent MYB repetitions, the MYB domain of each type of MYB TFs varied. At the same time, many conserved amino acids were also detected, especially for the tryptophan residues (W) (**Figure 1**). In the R2 and R3 domains of the R2R3-MYB TFs, there were three conserved W in R2 and only two conserved W in R3; the first W of R3 was replaced by phenylalanine (F) in this study (**Figure 1**).

### 3.4 Phylogenetic analysis of *Ipomoea* MYB proteins

**Figure 2:**
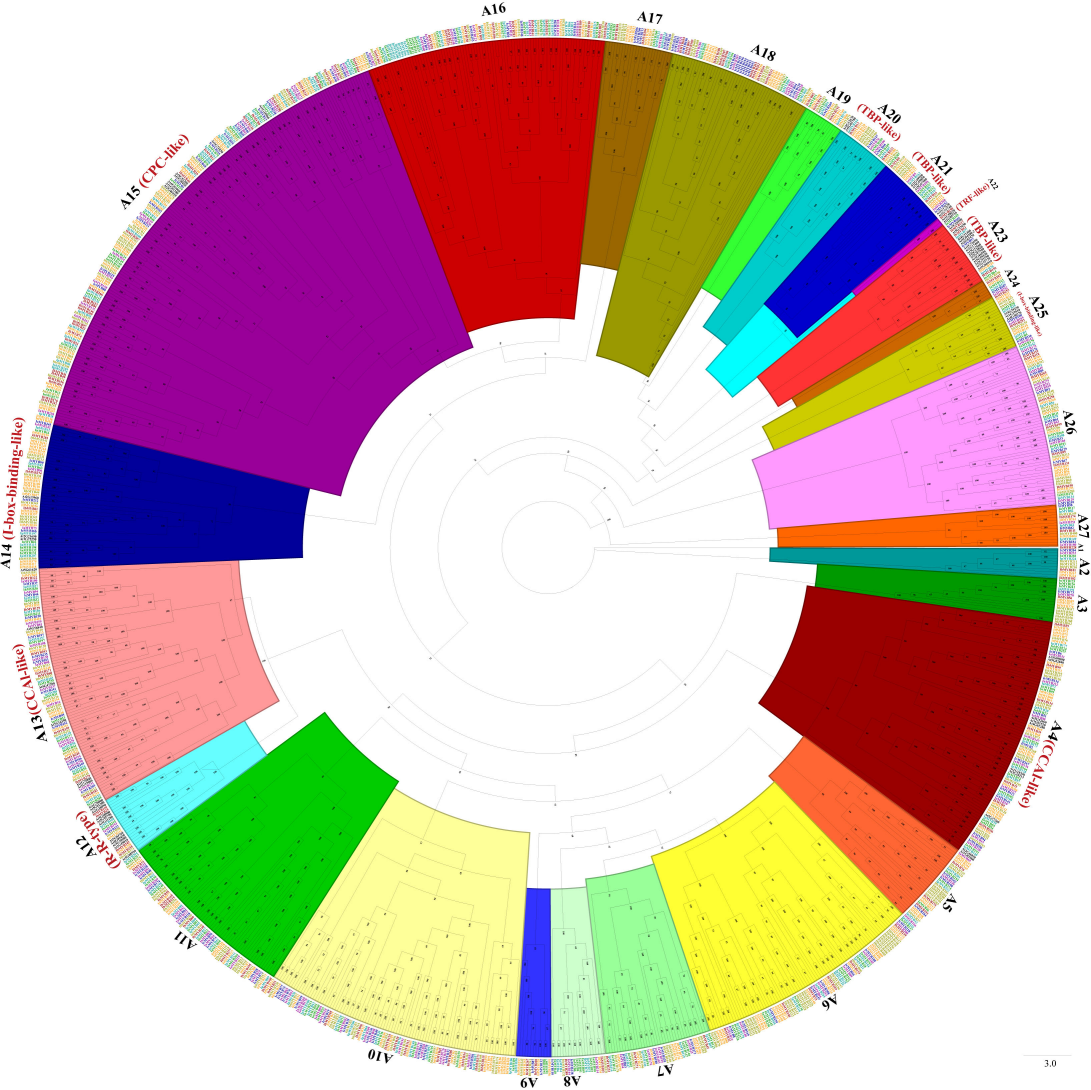
The phylogenetic tree of *Ipomoea* species and Arabidopsis MYB-related proteins. The proteins were grouped into 27 subgroups, each given a number (e.g., A1 to A27). The “CCA1-like,” “CPC-like,” “I-box-binding-like,” “R-R-type,” “TBP-like,” and “TRF-like” subfamilies were indicated with red name, respectively (Chen et al., 2006).

**Figure 3:**
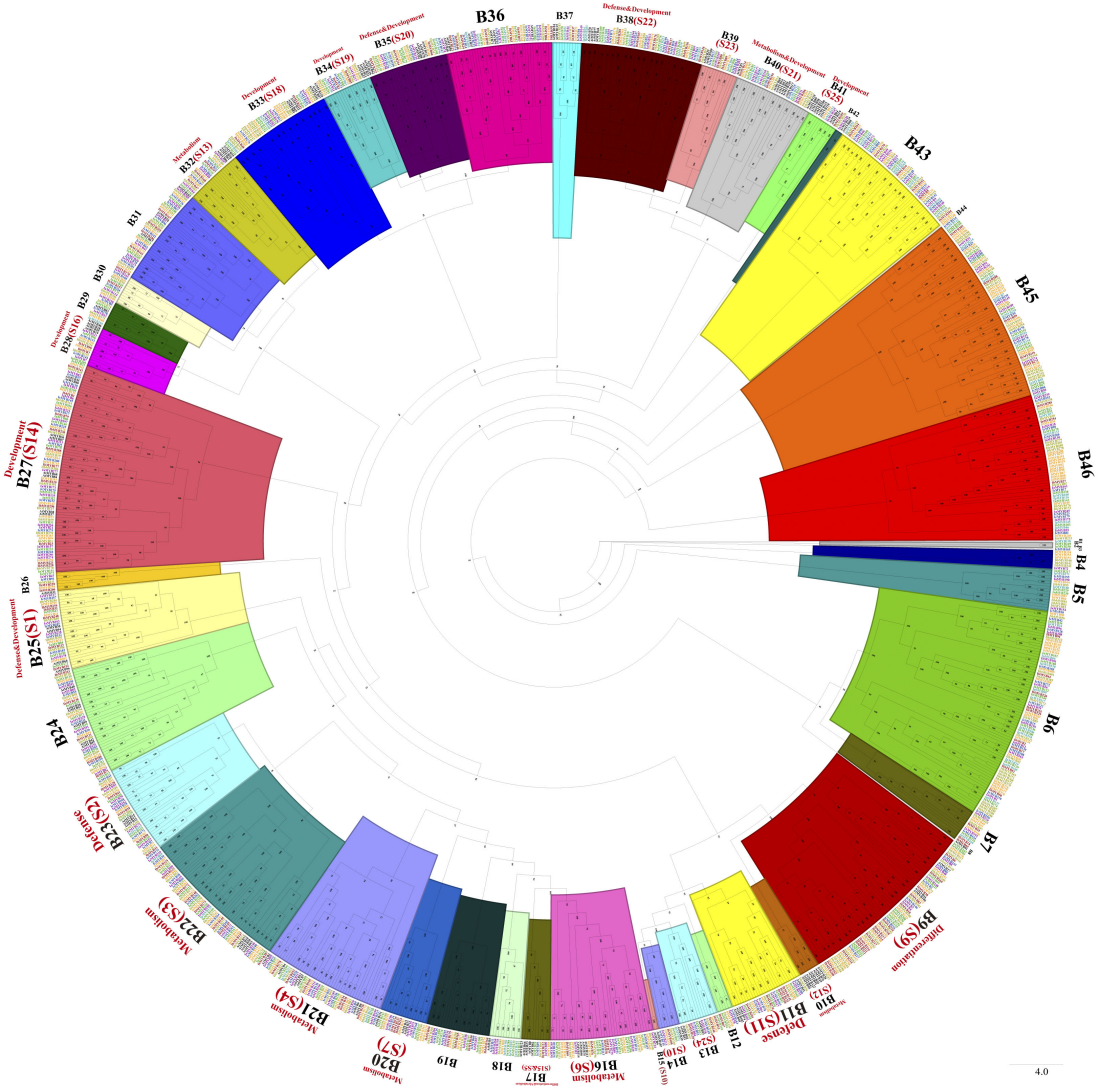
The phylogenetic tree of *Ipomoea* species and Arabidopsis R2R3-MYB proteins. The proteins were grouped into 46 subgroups, and each group has given a number (e.g., B1 to B46). S1–S25 indicated the Arabidopsis subfamily identified previously and were written in red words, as well as the prospective functions of the Arabidopsis MYB TFs in each subfamily (Dubos et al., 2010).

Phylogenetic trees were constructed with MYB-related TFs, R2R3-MYB TFs, 3R-MYB TFs, and the other (4R- and 5R-MYB) TFs of *Ipomoea* species and Arabidopsis, respectively. Based on the tree’s topology, the MYB-related TFs of *Ipomoea* species and Arabidopsis were classed into 27 subgroups, from A1 to A27 (**Figure 2**). The Arabidopsis MYB-related TFs dispersed in 10 of the 27 subgroups (i.e., A4, A12, A13, A14, A15, A20, A21, A22, A23, and A25) (**Figure 2**). According to the classification of Arabidopsis MYB-related TFs (Chen et al., 2006), we identified the CCA1-like subfamily (A4 and A13), R-R-type subfamily (A12), I-box-binding-like subfamily (A14 and A25), CPC-like subfamily (A15), TBP-like subfamily (A20, A21and A23), and TRF-like subfamily (A22) (**Figure 2**). The R2R3-MYB TFs of *Ipomoea* species and Arabidopsis were divided into 46 subgroups, from B1 to B46 (**Figure 3**). Based on the previous study of Arabidopsis R2R3-MYB TFs (Dubos et al., 2010), the S1 to S25 subfamilies and potential functions of the genes in each subfamily are indicated in **Figure 3**; for instance, B11 and B23 were indicated as S11 and S2, respectively, and the gene in the two subgroups might be involved in defense function (**Figure 3**). The 3R-MYB TFs were classed into 10 subgroups (C1 to C10). All of the 3R-MYB TFs of sweet potato, except for *IbMYB275*, were clustered together in subgroup C10 (**Figure S2**). The 3R-MYB TFs belonging to the same species tended to cluster together, for example, subgroups C3, C4, C7, and C10. In comparison, the genes clustered in the subgroups C2, C6, and C8 were always from different species (**Figure S2**). The results indicated that the members of *Ipomoea* 3R-MYB TFs might experience different evolutions after the species differentiation: some kept the initial copy number of their ancestors, whereas others increased their copies (**Figure S2**). The 4R- and 5R-MYB TFs of the seven *Ipomoea* species were divided into four subgroups, named D1 to D4 (**Figure S3**).

### 3.5 Gene structures and motif composition of *MYB* genes in *Ipomoea* species

As shown in **Figure S4**, the gene’s structural diversity might also be a reflection of the evolutional divergence among homologous *MYB* genes. In other words, the *Ipomoea MYB* genes clustered in the same subgroups shared a roughly similar exon/intron structure, exon number, and gene length (**Figure S4**). The *MYB-related* genes harbored more exons and longer sequence lengths than R2R3-MYB genes (**Figure S4**, **Table S3**). The distributions of motifs of *MYB* proteins in the same subgroup were also roughly similar, suggesting that the motif distributions might be related to functions (**Figure S4**). The motifs that consist of the core *MYB* domain were the most conservative: for the *MYB-related* genes, motif 2 was the most conserved, followed by motif 3, motif 1, and motif 8; for the *R2R3-MYB* genes, motif 3 was the most conserved, followed by motif 2, motif 6, and motif 12; 19 of the 23 *3R-MYB* genes contained motif 1 and motif 15; and all of the *4R-* and *5R-MYB* genes contained motif 1 and motif 2 (**Figure S4**, **Table S6**).

### 3.6 Chromosomal location and duplication analysis of the *Ipomoea MYB* genes

**Figure 4:**
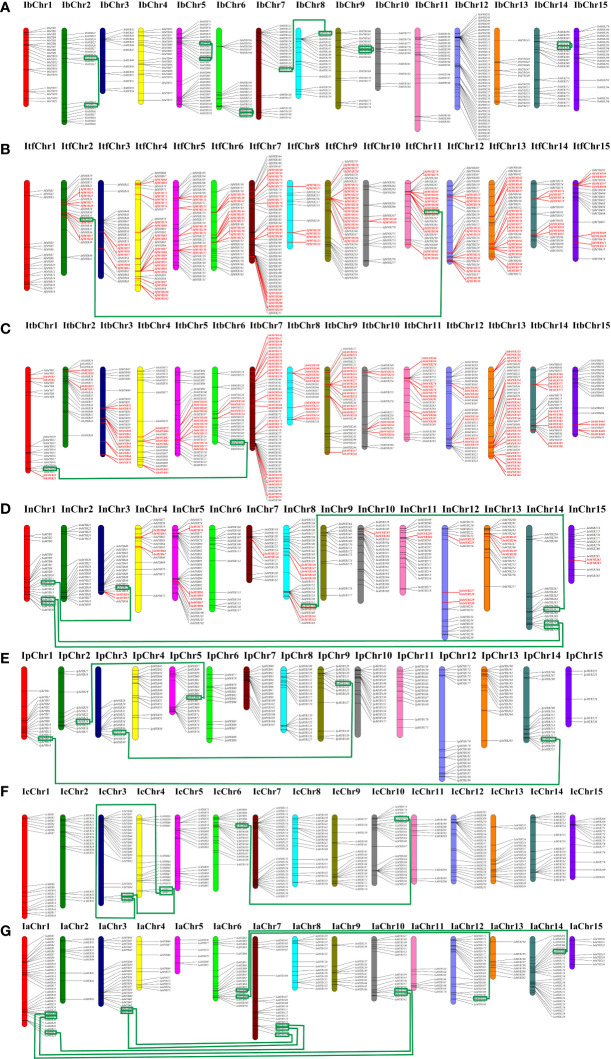
Distribution of *MYB* genes across the chromosomes of the seven *Ipomoea* species. **(A)** Distribution in sweet potato (*I. batatas*). **(B)** Distribution in (*I.*) *trifida*. **(C)** Distribution in (*I.*) *triloba*. **(D)** Distribution in (*I.*) *nil*. **(E)** Distribution in (*I.*) *purpurea*. **(F)** Distribution in (*I.*) *cairica*. **(G)** Distribution in (*I.*) *aquatic*. The red color indicates the tandemly duplicated *MYB* genes on the chromosomal positions; the green line connected green square frame indicated segmentally duplicated genes.

To understand the genomic distribution of the *Ipomoea MYB* genes, chromosomal location analysis was performed. As shown in **Figure 4**, the *Ipomoea MYB* genes were unevenly distributed across the chromosomes of the *Ipomoea* species (**Figure 4**). Duplication analysis showed that 106, 105, and 20 pairs of tandemly duplicated *MYB* genes were found in *I. trifida*, *I. triloba*, and *I. nil*, respectively. In contrast, no duplicated *MYB* genes were detected in sweet potato (*I. batatas*), *I. purpurea*, *I. cairica*, and *I. aquatic* (**Figure 4**, **Table S7**). Meanwhile, a total of six, one, one, four, three, three, and seven pairs of segmentally duplicated genes were found in sweet potato (*I. batatas*), *I. trifida*, *I. triloba*, *I. nil*, *I. purpurea*, *I. cairica*, and *I. aquatic*, respectively (**Figure 4**, **Table S7**). These results suggested that both segmental and tandem duplications have played essential roles in *MYB* gene expansion in *I. trifida*, *I. triloba*, and *I. nil*, and tandem duplications were predominant, whereas in sweet potato (*I. batatas*), *I. purpurea*, *I. cairica*, and *I. aquatic*, segmental duplications were predominant.

### 3.7 Syntenic analysis of *MYB* genes in the genomes of *Ipomoea* species

**Figure 5:**
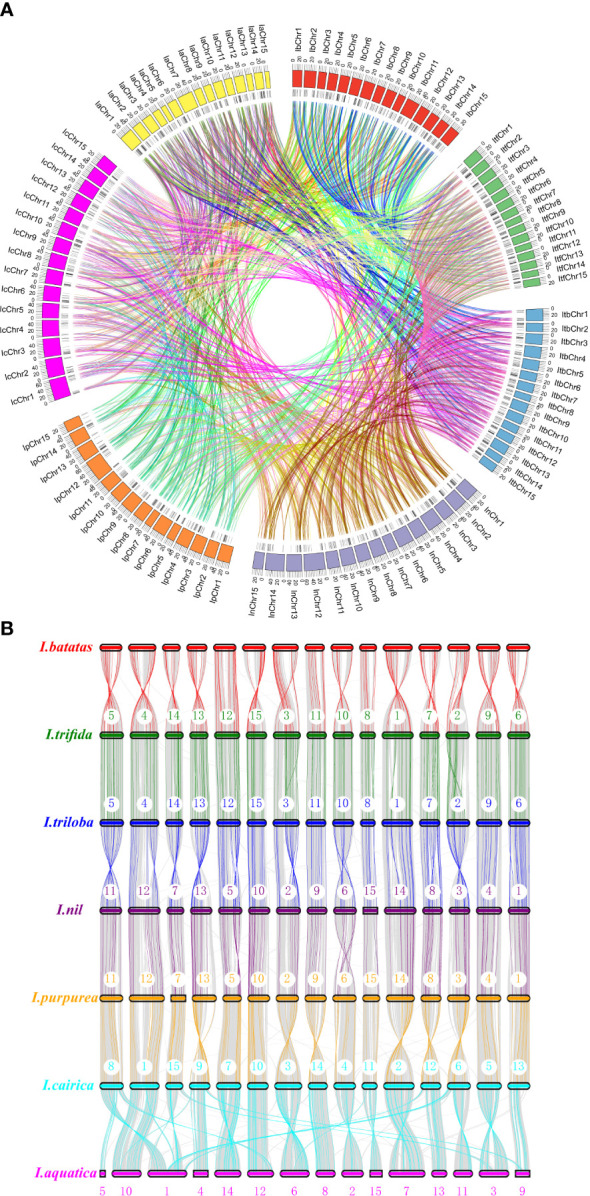
Syntenic analyses of *MYB* genes in the genomes of *Ipomoea* species. **(A)** Chromosomal distribution in the seven *Ipomoea* species. The outer circle represents the haploid chromosomes of sweet potato (*I. batatas*) (red), (*I.*) *trifida* (green), *(I.) triloba* (cornflower blue), (*I.*) *nil* (medium purple), (*I.*) *purpurea* (orange), (*I.*) *cairica* (magenta), and (*I.*) *aquatic* (yellow), respectively. The second circle (black) represents the matches of *MYB* genes with the genome of the *Ipomoea* species. Colorful lines show the collinear *MYB* gene pairs in the whole genome of the *Ipomoea* species. **(B)** Schematic representation of syntenic genes among sweet potato (*I. batatas*), (*I*) *trifida*, *(I) triloba*, (*I*) *nil*, (*I*) *purpurea*, (*I*) *cairica*, and (*I*) *aquatic*. The chromosomes of the seven Ipomoea species were reordered. Gray lines in the background indicate the collinear blocks within *Ipomoea* genomes, with *MYB* gene pairs highlighted in chromatic color. The black lines indicated synteny gene pairs between the seven *Ipomea* species.

To determine the evolutionary mechanism of *Ipomoea MYB* genes, comparative synteny maps of the seven *Ipomoea* species were constructed (**Figure 5**). A total of 1,703 *Ipomoea MYB* genes that formed 4,050 ortholog pairs were detected in the seven *Ipomoea* species (**Tables S8**, **S9**). Of these genes, *R2R3-MYB* genes were the most (890), followed by *MYB-relative* (782), *3R-MYB* (24), and *5R-MYB* (7) genes; when referring to subfamilies, the A15 members were the most (115), followed by the A10 (77), A13 (71), A16 (67), B27 (66), A4 (66), B6 (61), and other subfamily (<60) members (**Tables S8**, **S9**). *I. trifida* and *I. triloba* harbored the most ortholog *MYB* gene pairs (248 pairs), followed by *I. triloba* and *I. cairica* (240 pairs), *I. nil* and *I. cairica* (223 pairs), and *I. trifida*, and *I. cairica* (221 pairs) (**Table S8**). The sweet potato showed more than 200 ortholog *MYB* gene pairs with *I. triloba* (214 pairs), *I. cairica* (214 pairs), and *I. trifida* (208 pairs) (**Table S8**). The ortholog *MYB* genes were distributed in all of the subfamilies except for A1, A22, and D1 (**Table S8**). In most cases (3,636 of 4,150, 87.61%), two of the ortholog *MYB* genes were from the same subfamily (**Table S10**). The A13 subfamily held the most *MYB* gene pairs (166 pairs), followed by A10 (161 pairs), A4 (161 pairs), A16 (156 pairs), B6 (145 pairs), and B45 (134 pairs) (**Table S10**). A total of 621 *MYB* genes (90 from sweet potato, 86 from *I. trifida*, 90 from *I. triloba*, 91 from *I. nil*, 87 from *I. purpurea*, 89 from *I. cairica*, and 88 from *I. aquatic*) can be traced to form ortholog gene pairs between any two of the seven *Ipomea* species (**Figure 5**, **Table S11**). Of the 621 *MYB* genes, the A4 subfamily members were the most abundant (40), followed by the A16 (36), B6 (36), A15 (33), A10 (30), B45 (29), B43 (24), A13 (23), and other (<20) subfamily members (**Table S11**).

### 3.8 Ka/Ks analysis of duplicated and syntenic *MYB* genes

To detect whether some *MYB* genes are under positive selection, Ka/Ks analysis was performed on duplicated and syntenic *MYB* genes within or between the seven *Ipomoea* species. Within the seven *Ipomoea* species, all of the segmentally duplicated genes (23 pairs) harbored a Ka/Ks ratio <1, suggesting that these genes underwent purifying (negative) evolutionary selection. Fifty-one pairs of tandemly duplicated genes’ Ka/Ks <1 underwent purifying (negative) evolutionary selection, and 21 pairs of tandemly duplicated genes possessed Ka/Ks ratio >1 and were subjected to positive selection (**Table S12**). Between the seven *Ipomoea* species, 99.5% (4,116 pairs) of the syntenic *MYB* genes possessed Ka/Ks ratio <1 and the other ones (0.5%, 22 pairs) possessed Ka/Ks ratio >1 (**Table S12**). These results suggest that the majority of duplicated and syntenic *MYB* genes were subjected to purifying selection inside duplicated genomic elements during speciation. In contrast, a smaller number of such genes were subjected to positive selection.

### 3.9 *Cis*-regulatory elements (CREs) in putative promoter regions of the *Ipomoea MYB* genes

A 1.5-kb upstream promoter region of *Ipomoea MYB* genes was scanned for CRE analysis. A lot of CREs related to plant growth and development (light response, cell cycle, endosperm expression, meristem expression, root-specific and seed-specific regulation, and zein metabolism regulation) and stress response (ABA response, anaerobic induction, defense and stress responsiveness, drought stress, flavonoid biosynthetic genes regulation, gibberellin responsiveness, SA responsiveness, low-temperature responsiveness, and MeJA response) were detected (**Figure S5**, **Table S13**). Of the CREs related to plant growth and development, the Box 4 motif was the most abundant, followed by the G-Box motif and GT1-motif, and all of them contributed to light response. Of the CREs related to stress response, ABRE motif (ABA response) was the most abundant, followed by ARE (anaerobic induction), TGACG-motif (MeJA response), MBS (drought stress), TCA-element (SA responsive), LTR (low-temperature responsiveness), TGA-element (anaerobic induction), and TC-rich repeats (defense and stress responsiveness) (**Figure S5**, **Table S13**).

The entire CREs shown in the head of **Table S13** appeared in both *MYB-related* and *R2R3-MYB* promoter regions, with Box 4 being the most abundant, followed by G-Box, ABRE, and ARE (**Table S14**). However, some CREs (3-AF1-binding site, AAAC-motif, chs-Unit 1 m1, GATT-motif, motif I, RY-element, TGA-box, and RY-element were detected in *3R-MYB* promoter regions and the AAAC-motif, AT1-motif, ATC-motif, ATCT-motif, CAG-motif, chs-CMA2a, chs-Unit 1 m1, GA-motif, Gap-box, GATT-motif, GTGGC-motif, LS7, MRE, MSA-like, motif I, RY-element, TGA-box, TC-rich repeats, and MBSI) were absent in *3R-*, *4R-*, and *5R-MYB* promoter regions (**Table S14**). The CGTCA-motif, ABRE, ARE, LTR, and TGACG-motif were the most conserved in the *Ipomoea MYB-related* promoter regions, and they could be detected in all of the subfamilies of this type of genes (**Table S14**). Box 4 was the most conserved in the *Ipomoea R2R3-MYB* promoter regions, followed by TCT-motif, CGTCA-motif, ARE, and TGACG-motif, and they were absent in no more than three of the subfamilies of this type of genes (**Table S14**). Box 4 and ARE were the most conserved in the subfamilies of *3R-MYB*, and chs-CMA1a, G-Box, and ABRE were the most conserved in the subfamilies of *4R-* and *5R-MYB* (**Table S14**). However, some CREs showed subfamily specificity; for example, the GATT motif was only detected in the A13, A14, A15, A18, B20, B22, and B37 subfamilies (**Table S14**). In each subfamily of the MYB promoter regions, the distribution of the CREs was not conservative as the gene-coding sequences; the distribution even showed gene specificity (**Figure S6**). The same distribution of the CREs occasionally appeared in tandem-duplicated genes; for example, *ItbMYB210*, *ItbMYB211*, and *ItbMYB212* were tandem-duplicated genes detected in *I. triloba*, and they shared the same CRE distribution. However, the MYB genes in the same family may show similarity in the number and type of CREs; for instance, *ItfMYB68*, *IbMYB39*, and *ItfMYB69* in the A4 subfamily, and *ItbMYB45*, *IbMYB141*, and *ItfMYB42* in the B27 subfamily (**Table S13**; **Figure S6**).

### 3.10 Expression profiles of *MYB* genes in sweet potato

**Figure 6:**
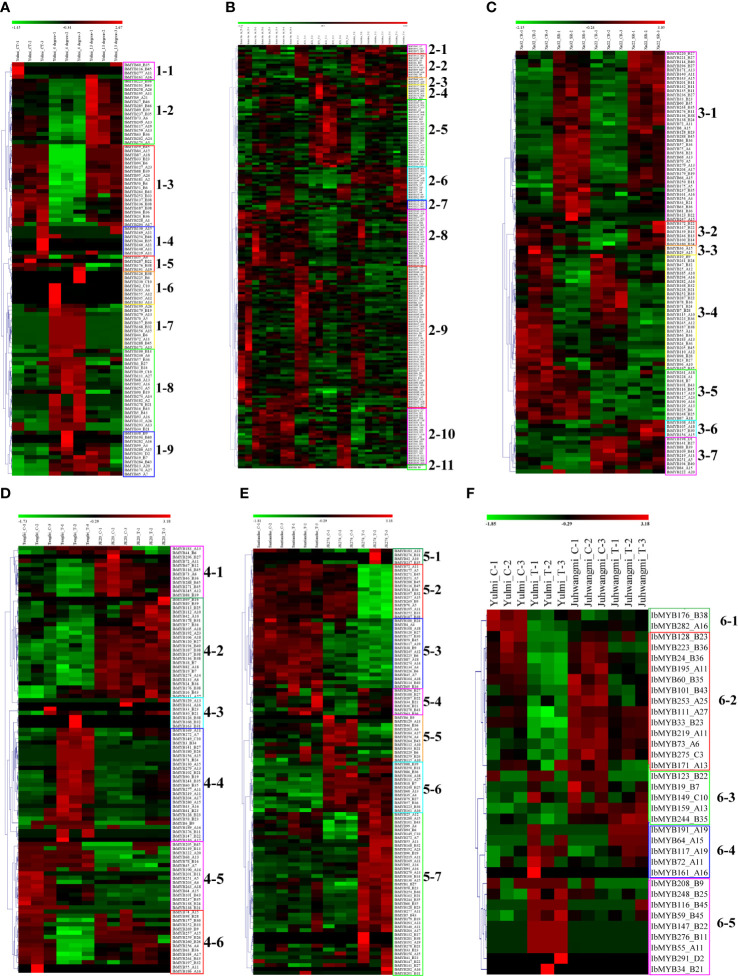
Heatmap of the expression profiles of sweet potato *MYB* differentially expressed genes (DEGs) in response to abiotic and biotic stresses. **(A)** *MYB* DEGs in “Yulmi” under control (CT), 4°C, and 13°C. **(B)** *MYB* DEGs in “Kokei 14,” “KT 1,” and “*I. triloba*” during different times of drought treatments. **(C)** *MYB* DEGs in “Xu 32” and “Xu 22” under control and salt treatments. CR, control; SR, treatment. **(D)** *MYB* DEGs in “Tengfei” and “JK20” under control and sweet potato stem nematode inoculation. C, control; T, treatment. **(E)** *MYB* DEGs in “Santiandao” and “JK274” under control and *Ceratocystis fimbriata* inoculation. C, control; T, treatment. **(F)** *MYB* DEGs in “Yulmi” and “Juhwangmi” under control and root-knot nematode inoculation. C, control; T, treatment. The letters after the MYB gene name indicated its phylogenetic subgroup.

To explore *MYB* genes related to stress response, six transcriptome datasets referring to abiotic stresses (cold, drought, and salt) and biotic stresses (sweet potato stem nematodes, *Ceratocystis fimbriata*, and root-knot nematodes) were analyzed. A total of 101 *MYB* differentially expressed genes (DEGs) were detected in the transcriptome dataset of chilling injury at low-temperature storage, which was further classed into nine subclasses (1–1 to 1–9) based on their expression (**Figure 6A**). Compared with the control temperature (CT), the *MYB* genes in subclass 1-2 were upregulated at 13°C and downregulated at 4°C; the ones in subclass 1-3 were unchanged at 13°C and downregulated at 4°C; the ones in subclasses 1-7 and 1-8 were all unchanged at 13°C and upregulated at 4°C (**Figure 6A**). A total of 180 *MYB* DEGs were found in the drought stress transcriptome analysis, and they were classed into 11 subclasses (2-1 to 2-11) (**Figure 6B**). With the extension of drought time, the *MYB* genes in subclass 2-5 mainly maintained high expression in drought sense cultivar “Kokei 14,” whereas they were comparably downregulated in both “KT 1” and “*I. triloba*” (drought-tolerant types) (**Figure 6B**). A total of 102 MYB DEGs were found in the salt stress transcriptome analysis, and they were classed into seven subclasses (3-1 to 3-7) (**Figure 6C**). Compared with the control, the *MYB* genes in subclass 3-1 were upregulated in both “Xu 32” (salt sense cultivar) and “Xu 22” (salt-tolerant cultivar), whereas the *MYB* genes in subclass 3-4 were downregulated in the two investigated cultivars; compared with the salt-sensitive cultivar “Xu 32,” the *MYB* genes in subclass 3-5 were kept downregulated under control and salt treatment of “Xu 22”; conversely, the *MYB* genes in subclass 3-7 were mainly kept upregulated under control and salt treatment on “Xu 22” (**Figure 6C**).

A total of 110 *MYB* DEGs were found in the dataset of sweet potato stem nematode inoculation transcriptome analysis, and they were further classed into six subclasses (4-1 to 4-6) (**Figure 6D**). Compared with the sense cultivar “Tengfei,” the *MYB* genes in subclass 4-2 were mainly kept upregulated under control and sweet potato stem nematode inoculation of “JK20” (sweet potato line); compared to the control, the *MYB* genes in subclass 4-4 were upregulated in sweet potato stem nematode inoculation of “Tengfei,” whereas they remained downregulated in the same treatment of “JK20” (**Figure 6D**). There were 101 *MYB* DEGs found in the dataset of *Ceratocystis fimbriata* inoculation transcriptome analysis, and they were classed into seven subclasses (5-1 to 5-7) (**Figure 6E**). Compared with the control, the *MYB* genes in subclass 5-2 were downregulated in the *Ceratocystis fimbriata*-resistant line “JK274” after inoculation, whereas they kept a high expression in the sense cultivar “Santiandao”; the *MYB* genes in subclass 5-7 were upregulated in the *Ceratocystis fimbriata*-resistant line “JK274” after inoculation, whereas they kept a low expression in the sensitive cultivar “Santiandao”; the *MYB* genes in subclass 5-3 kept a high expression in “Santiandao” and a low expression in “JK274”; and the *MYB* genes in subclass 5-6 kept a low expression in “Santiandao” and a high expression in “JK274” (**Figure 6E**). There were 34 *MYB* DEGs found in the dataset of root-knot nematode inoculation transcriptome analysis, and they were classed into five subclasses (6-1 to 6-5) (**Figure 6F**).

**Figure 7:**
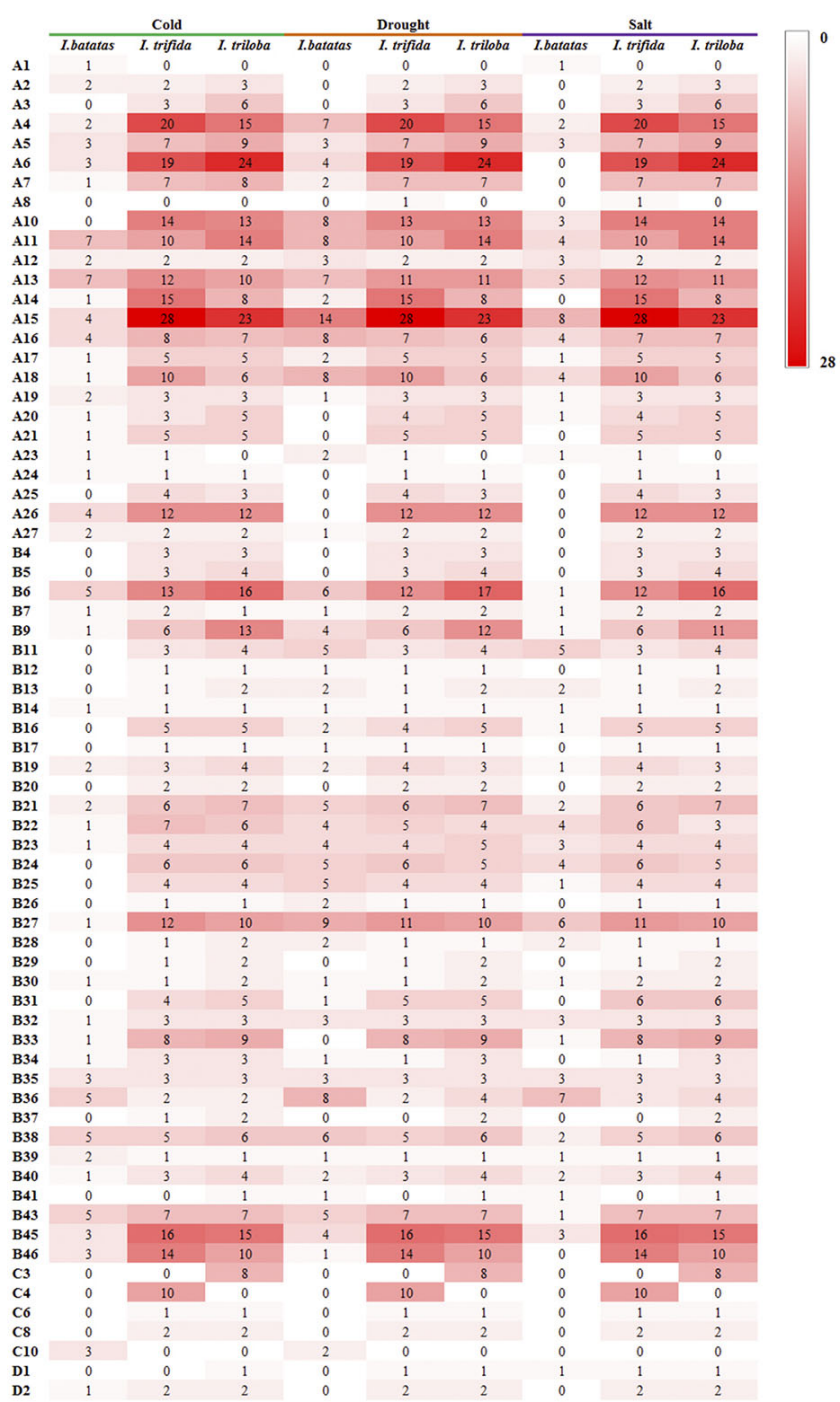
Comparison of subfamily-based gene numbers under abiotic stresses in the sweet potato, *I. trifida*, and *I. triloba*.

During the drought and salt treatments of sweet potato, the DEGs belonging to the A15, B27, and B36 phylogenetic subfamily were the most, whereas in cold stress, the DEGs belonging to the A11, A13, B38, B43, B6, and B36 subfamilies were the most (**Figures 6** ,**7**). During the biotic stress treatments, the DEGs belonging to the A11, A13, A16, and B23 subfamilies were abundant (**Figures 6**, **7**). Both the abiotic and biotic stress treatments detected DEGs in the B11, B23, B25, B35, and B38 subfamilies, the members of which were considered to have the function of defense (**Figures 3**, **6**, **7**). To compare the transcriptional changes of various *MYB* subfamilies between species, the *I. trifida* and *I. triloba MYB* genes that responded to abiotic stresses were analyzed. During the cold, drought, and salt treatments, 365, 359, and 366 *I. trifida MYB* DEGs and 370, 369, and 368 *I. triloba MYB* DEGs were found, respectively (**Tables S15**, **S16**). When comparing the results with that of sweet potato, we discovered that the *MYB* DEGs of the three *Ipomoea* species dispersed in 68 phylogenetic subfamilies (**Figure 7**). Of these subfamilies, 41, 45, and 38 were common in the three *Ipomoea* species and contained DEG response to cold, drought, and salt treatments, respectively (**Figure 7**, **Table S17**). Further analysis revealed that the *MYB* DEG response to abiotic stresses generally belonged to MYB-related and R2R3-MYB gene types, involving 28 phylogenetic subfamilies, such as A4, A5, A13, A15, B23, B35, and B38 (**Figure 7**, **Table S18**).

### 3.11 Expression analysis of sweet potato *MYB* genes by quantitative reverse-transcription polymerase chain reaction (qRT-PCR)

Based on transcriptome results, *IbMYB68*, *IbMYB237*, *IbMYB39*, *IbMYB128*, *IbMYB127*, *IbMYB201*, *IbMYB82*, *IbMYB187*, *IbMYB4*, and *IbMYB18* were selected for further analysis using qRT-PCR. Under cold treatments, compared with the control condition (0 h), the transcripts of *IbMYB68* in “JK328” were upregulated and peaked at 12 h after treatments, whereas there was no obvious change in “Xu 32”; the transcripts of *IbMYB237* in both “Xu 32” and “JK328” were upregulated and peaked at 6 and 24 h after treatments, respectively (**Figure 7**). Under drought treatments, compared with the control condition (0 h), the transcripts of *IbMYB39* and *IbMYB128* in “Xu 32” were upregulated and peaked at 12 and 4 h after treatments, respectively, whereas their transcripts showed no obvious change in “JK328” (**Figure 7**). Under salt treatments, compared with the control condition (0 h), the transcripts of *IbMYB127* in “Xu 32” were upregulated and peaked at 12 h after treatments, whereas the transcripts of *IbMYB127* in “JK328” were downregulated and decreased to valley at 12 h after treatments; the transcripts of *IbMYB201* in both “Xu 32” and “JK328” were upregulated and peaked at 6 and 12 h after treatments, respectively (**Figure 7**). Under sweet potato stem nematode infection treatments, compared with the control condition (0 h), the transcripts of *IbMYB82* in both “Tengfei” and “JK20” were upregulated and decreased to the valley at 2 days and 12 h after treatments, respectively; the transcripts of *IbMYB187* in “Tengfei” were downregulated and peaked at 1 day after treatments, whereas the transcripts of *IbMYB187* in “JK20” were upregulated and peaked at 12 h after treatments (**Figure 7**). Under *Ceratocystis fimbriata* inoculation treatments, compared with the control condition (0 h), the transcripts of *IbMYB4* and *IbMYB18* in “JK274” were upregulated and peaked at 2 days and 1 day hours after treatments, respectively, whereas their transcripts showed no obvious changing in “JK274” (**Figure 7**).

## 4 Discussion

The MYB transcription factor gene family is one of plants’ most prominent transcription factor families. It is involved in various essential life processes, including plant growth (Zhao et al., 2019), development (Liu et al., 2021; Yu et al., 2021), biosynthesis (Sinaga et al., 2021), and defense responses (Dubos et al., 2010; Lv et al., 2021; Hawku et al., 2022). Therefore, this gene family attracted more and more attention from various plant researchers and has been extensively investigated in many plant species, such as Arabidopsis (Chen et al., 2006), rice (Chen et al., 2006), soybean (Du et al., 2012b), maize (Du et al., 2012a), and potato (Li et al., 2021c). The *Ipomoea* contains 600–700 species, many of which are of considerable importance as medicinal or ornamental plants (Nimmakayala et al., 2011). The sweet potato is a highly heterozygous hexaploid (2n = 6x = 90) with a large, complex genome approximately 2–3 Gb in size (Ozias-Akins and Jarret, 1994). It is the world’s seventh most important food crop by production quantity (https://www.fao.org/faostat/, accessed 2022 November). *I. trifida* and *I. triloba* are two diploid species closely related to the hexaploid sweet potato, with an estimated genome size of 526.4 and 495.9 Mb, respectively (Wu et al., 2018). Due to their smaller genome size and ploidy, they were generally used as model plants to facilitate sweet potato breeding. *I. nil* is an annual climbing herb-producing blue flower capable of self-pollination (Hoshino et al., 2016). With an estimated genome size of 750 Mb, it has been a model plant for studying photoperiodic flowering and flower coloration. *I. purpurea*, with an estimated haploid genome size of 814 Mb, is a common agricultural weed exhibiting varying levels of herbicide resistance (Gupta et al., 2021). *I. cairica* is a perennial creeper introduced as a garden ornamental, and it has an estimated haploid genome size of 730 Mb (Jiang et al., 2022). *I. aquatica*, with an estimated genome size of 550.03 Mb, is usually called water spinach (Hao et al., 2021). It is one of the most popular green leafy vegetables, with aquatic and terrestrial characteristics (Hao et al., 2021). However, little is known about the MYB gene family in *Ipomoea* species.

In the present study, a total of 2,212 MYB genes were identified from the seven *Ipomoea* species, and the method used to identify the *Ipomoea MYB* genes was reliable and has been reported by previous studies (Zhou et al., 2020; Li et al., 2021c; Wang et al., 2022). Significant variations in *MYB* family members were observed in our analyzed *Ipomoea* species. There were 296, 430, 411, 291, 226, 281, and 277 *MYB* genes identified from sweet potato (*I. batatas*), *I. trifida*, *I. triloba*, *I. nil*, *I. purpurea*, *I. cairica*, and *I. aquatic*, respectively (**Tables S1**, **S2**). The number of MYB TFs in the seven investigated *Ipomoea* species differed, suggesting that *MYBs* in different *Ipomoea* plants have expanded to different degrees (Cao et al., 2021). By comparing the genome size and the number of identified *MYB* genes in this study, it is found that the number of *MYB* genes in each *Ipomoea* species was not highly correlated with the genome size (Yang et al., 2019; Liu et al., 2020a). Moreover, it might also correlate with the quality of the assembled *Ipomoea* genomes, and the MYB gene number will change along with the improvement of *Ipomoea* genome quality (Yang et al., 2019; Liu et al., 2020a). In various plants, the number of *MYB* genes is different; for instance, 198 MYBs were identified in Arabidopsis (Chen et al., 2006), 253 in *Hedychium coronarium* (Abbas et al., 2021), 235 in *Capsicum* spp. (Arce-Rodríguez et al., 2021), 174 in *M. rubra* (Cao et al., 2021), and 155 in *Petunia axillaris* (Chen et al., 2021). A similar phenomenon has also been reported in other closely related species, for instance, *S. lycopersicum* (127) and *S. tuberosum* (217) (Li et al., 2016; Li et al., 2021c), *M. acuminate* (305), and *M. balbisiana* (251) (Tan et al., 2020). The identified *Ipomoea* MYB TFs were classed into five types: MYB-related (1R-MYB), R2R3-MYB (2R-MYB), 3R-MYB, 4R-MYB, and 5R-MYB. Among these types, the MYB-related or R2R3-MYB was the largest in *Ipomoea* species, and they contain the highest quantity of genes within the MYB family. The R2R3-MYB was usually the largest type in green plants (Martin and Paz-Ares, 1997; Abbas et al., 2021; Arce-Rodríguez et al., 2021; Wang et al., 2021b; Li et al., 2021c), whereas the MYB-related type was also reported as the largest subfamily in some species (Mmadi et al., 2017; Pu et al., 2020).

In order to investigate the evolutionary relationships of *Ipomoea MYB* genes, a systematic analysis was performed on MYB-related TFs, R2R3-MYB TFs, 3R-MYB TFs, and the other (4R- and 5R-MYB) TFs of *Ipomoea* species and Arabidopsis, and four trees of them were constructed, respectively. Based on the topology, the trees were further divided into several subgroups (**Figures 2**, **3**, **Figures S2**, **S3**). Supposed that genes belonging to the same branch may experience a standard evolutionary process and possess a conserved function (Li et al., 2021c), this study compared the *MYB* genes of *Ipomoea* species and Arabidopsis. Subfamilies containing the *MYB* members from the *Ipomoea* species, Arabidopsis, and some species-specific subfamilies were detected. Similar results have been reported by phylogenetic analysis of other species (Wang et al., 2021b; Li et al., 2021c). The phylogenetic trees of MYB protein constructed in this study would help to predict the function of *Ipomoea MYB* genes with Arabidopsis *MYB* genes as a reference (Chen et al., 2006; Dubos et al., 2010).

The *Ipomoea MYB* genes identified in this study were different in amino acid sequence length, molecular weight, and isoelectric point, reflecting the complexity and functional diversity of the *Ipomoea MYB* genes to some degrees centigrade (**Tables S2**-**5**). The structure analysis of the *Ipomoea MYB* genes was consistent with phylogenetic analysis. Most genes in the same subgroup exhibited similar exon–intron structures (**Figure S4**). The *Ipomoea* MYB proteins within the same subgroup showed similar motif compositions, whereas the ones in different subgroups were of high variance (**Figure S4**). This is consistent with previous reports of other species, such as *G. hirsutum* (Salih et al., 2016), *Prunus salicina* (Liu et al., 2020a), and *Helianthus annuus* (Li et al., 2020). It was also found that the motifs of the core *MYB* domain were the most conservative (**Figure 1**, **Figure S4**). The *Ipomoea MYB* genes with a close evolutionary relationship might have similar functions (Wang et al., 2021b). Conserved motifs within the same TF family, especially the ones in the same subfamilies, may play essential roles in protein-specific functions (Chen et al., 2021).

The chromosomal location showed that the *Ipomoea MYB* genes were unevenly distributed across the chromosomes of the *Ipomoea* species (**Figure 4**). The uneven distributions of *MYB* genes have also been detected in chromosomes of other species, such as *Jatropha curcas* (Zhou et al., 2015), *M. rubra* (Cao et al., 2021), and *C. equisetifolia* (Wang et al., 2021b). The gene duplication events, segmental and tandem duplication, played essential roles in gene family expansion and distribution of genes in plants (Cannon et al., 2004; Kong et al., 2007; Jiang et al., 2013). In the present study, segmental and tandem duplications contributed to the *MYB* gene expansion in *I. trifida*, *I. triloba*, and *I. nil*; tandem duplications were predominant, whereas in sweet potato (*I. batatas*), *I. purpurea*, *I. cairica*, and *I. aquatic*, segmental duplications were predominant (**Figure 4**, **Table S7**). The predominant types of duplications might be species-specific performance, for instance, in *H. coronarium* (Abbas et al., 2021), *Prunus salicina* (Liu et al., 2020a), and *Helianthus annuus* (Li et al., 2020), segmental duplications were dominant, whereas in watermelon (Qing et al., 2018) and palm (Zhou et al., 2020), tandem duplications were predominant.

To further explore the evolutionary relationships of *Ipomoea MYB* genes, the syntenic maps of the seven *Ipomoea* species were constructed and compared. There were 1,703 *Ipomoea MYB* genes that formed 4,050 ortholog pairs detected in the seven *Ipomoea* species (**Tables S8**, **S9**). The results showed that many *MYB* genes from *Ipomoea* species presented high levels of collinearity and suggested that these *MYB* genes probably come from a common ancestor (Liu et al., 2020a; Abbas et al., 2021). It was found that some *MYB* genes contained more than one counterpart in the *Ipomoea* species, suggesting that the MYB gene family might undergo different amplification in *Ipomoea* genomes (Liu et al., 2020a). The orthologous events occurred in all phylogenetic subfamilies except for the A1, A22, and D1 (**Table S8**). The A1 and A22 subfamily contained only one *Ipomoea MYB* gene (*IbMYB228* and *IbMYB290*) (**Figure 2**); the D1 subfamily consisted of three *MYB* members (*IbMYB198*, *ItfMYB414*, and *ItbMYB404*) (**Figure S3**). In most cases, the two *MYB* genes of orthologous pairs were generally from the same subfamily. The A13 subfamily held the most orthologous members, followed by A10, A4, A16, B6, and B45. A total of 621 *Ipomoea MYB* genes could form orthologous gene pairs between any two of the seven *Ipomea* species (**Figure 5**, **Table S11**).

Ka/Ks analysis of duplicated and syntenic *MYB* genes within or between the seven *Ipomoea* species showed that most duplicated and syntenic *MYB* genes were subjected to purifying selection inside duplicated genomic elements during speciation. In contrast, fewer such genes were subjected to positive selection (**Table S12**). These results indicated that *Ipomoea MYB* genes underwent a robust purifying selection during evolution with slight variation after duplication (Zhou et al., 2020). The phenomenon of the high proportion of purifying selection and low proportion of positive selection has been reported in previous MYB gene family studies, such as *G. hirsutum* (Salih et al., 2016), *Elaeis guineensis* (Zhou et al., 2020), and *H. coronarium* (Abbas et al., 2021).

The CREs in promoters of genes are usually involved in transcription initiation and regulation (Ho and Geisler, 2019; Mulat and Sinha, 2021). The present study scanned a 1.5-kb upstream promoter region of *Ipomoea MYB* genes for *cis*-regulatory elements analysis. Various CREs related to plant growth, development, and stress response were detected (**Figure S5**, **Table S13**). CREs related to stress response, such as ABRE, ARE, TGACG-motif, MBS, TCA-element, LTR, TGA-element, and TC-rich repeats, were abundant in the promoters of the *Ipomoea MYB* gene. Similar results have been reported by other species, such as *Medicago sativa* (Zhou et al., 2019), *H. coronarium* (Abbas et al., 2021), and *Petunia axillaris* (Chen et al., 2021). It was also found that some CREs were detected in *MYB-related* and *R2R3-MYB* promoter regions, whereas they were absent in *3R-*, *4R-*, and *5R-MYB* promoter regions, suggesting that different transcription initiation or regulation might occur between them (Ho and Geisler, 2019; Mulat and Sinha, 2021). In the subfamilies of *MYB-related* and *R2R3-MYB* genes, the CGTCA-motif, ABRE, ARE, and TGACG-motif were the most conserved. Moreover, some subfamily-specific CREs were also detected (**Table S14**). The distribution of the CREs was not conservative as the gene-coding sequences in each subfamily, and the non-conservative distribution of CREs has been reported by other TF gene families, such as NAC (Diao et al., 2018), WRKY (Zheng et al., 2021), and HSP20 (Cui et al., 2021). In this study, the exact distribution of the CREs occasionally appeared in tandemly duplicated genes. However, the *MYB* genes in the same family may show similarities in the number and type of CREs (**Table S13**; **Figure S6**).

**Figure 8:**
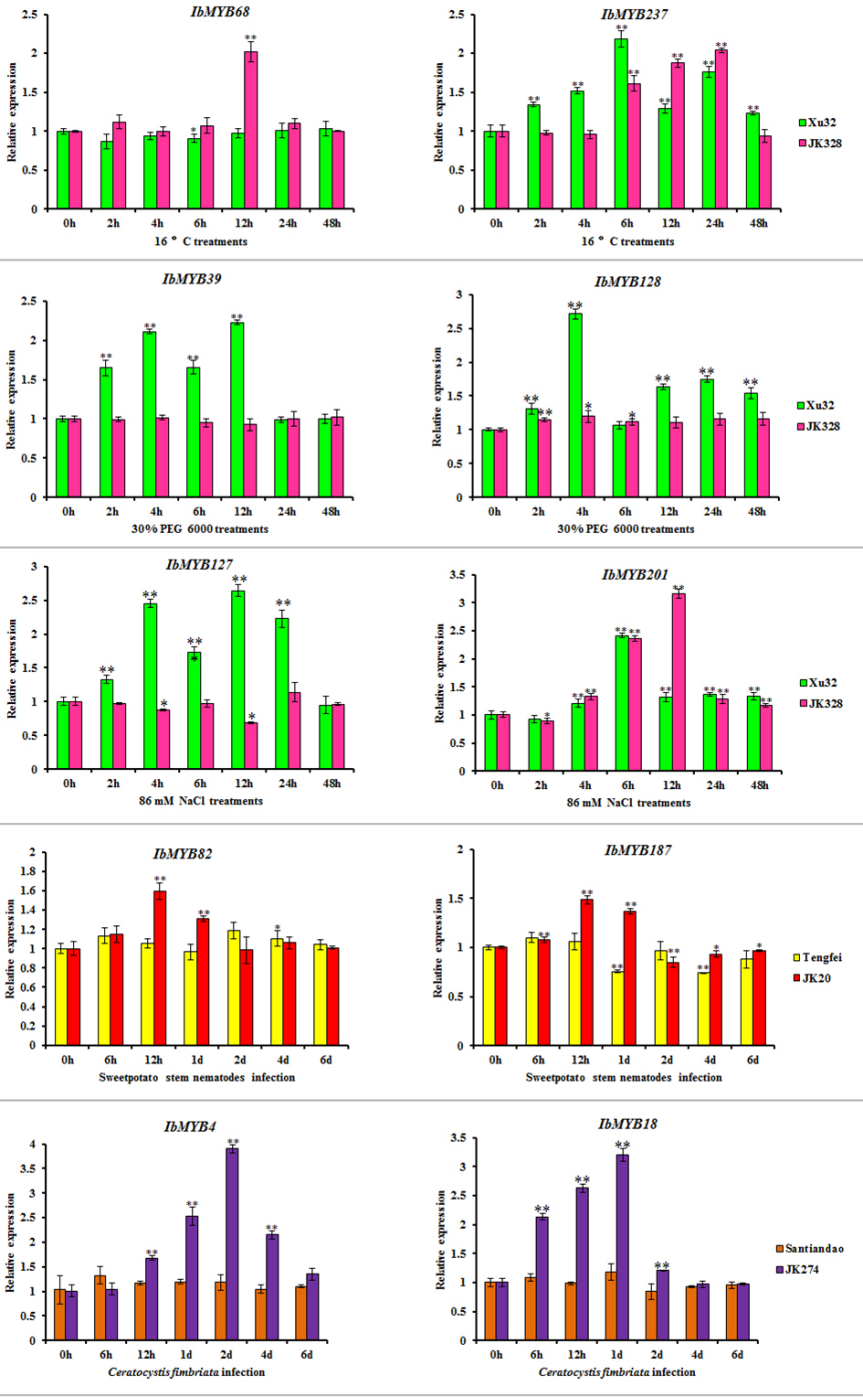
Expression analyses of *IbMYB* genes in sweet potato cultivars or lines. Denoted the significance of expression levels compared with control were ∗<0.05, ∗∗<0.01. h, hours; d, day(s).

Focused on the stress resistance of sweet potatoes, six transcriptome datasets referring to abiotic and biotic stresses were analyzed, and DEGs in each transcriptome dataset were detected. For comparative analysis, the *I. trifida* and *I. triloba MYB* genes that responded to abiotic stresses were also detected and analyzed in this study (**Figure 7**). The results showed that the *Ipomoea MYB* DEGs’ response to abiotic stresses usually belonged to MYB-related and R2R3-MYB types and was referred to various phylogenetic subfamilies (**Figure 7**, **Table S18**). In these subfamilies, some were considered as the defense function subfamilies with Arabidopsis MYB members as the reference (Chen et al., 2006), such as B11, B23, B25, B35, and B38 (**Figure 3**). During the abiotic stress treatments in the sweet potato, *I. trifida* and *I. triloba*, 28 phylogenetic subfamilies contained the *MYB* members that responded in all the abiotic stress treatments of the three *Ipomoea* species (**Figure 7**, **Table S18**). Based on the results of transcriptome datasets analysis, *IbMYB68*, *IbMYB237*, *IbMYB39*, *IbMYB128*, *IbMYB127*, *IbMYB201*, *IbMYB82*, *IbMYB187*, *IbMYB4*, and *IbMYB18* were selected for further analyzing using qRT-PCR (**Figure 8**). The results of qRT-PCR of the selected *IbMYB* genes were consistent with the transcriptome analysis. *IbMYB68* and *IbMYB237* responded to cold treatments, *IbMYB39* and *IbMYB128* responded to drought treatments, *IbMYB127* and *IbMYB201* responded to salt treatments, *IbMYB82* and *IbMYB187* responded to sweet potato stem nematode infection, and *IbMYB4* and *IbMYB18* responded to *Ceratocystis fimbriata* infection. These results indicated that the *IbMYB* genes were essential in biotic and abiotic stress responses.

## 5 Conclusions

This study is the first to systematically analyze the *MYB* gene family in *Ipomoea* species. There were 296, 430, 411, 291, 226, 281, and 277 *MYB* genes were identified from sweet potato (*I. batatas*), *I. trifida*, *I. triloba*, *I. nil*, *I. purpurea*, *I. cairica*, and *I. aquatic*, respectively. The identified *MYB* genes were classified into five types and were subjected to molecular weight (MW), isoelectric point (pI), and subcellular localization analysis. Based on the topology, phylogenetic analysis classed the *Ipomoea MYB* genes into several subgroups using the Arabidopsis *MYB* genes as a reference. Gene structure, motif organization, duplicated and syntenic analyses, and *cis*-regulatory element investigation were further performed. Focus on sweet potato, six transcriptome datasets referring to different abiotic and biotic stresses were analyzed and DEGs related to stress responses were detected. Based on the expression profiles of *IbMYBs*, 10 of the detected DEGs were selected for qRT-PCR analysis under five abiotic and biotic stress treatments. The results were consistent with the transcriptome analysis. This study may help to study the evolution of *MYB* genes in the *Ipomoea* species genomes and provide helpful information for the resistance breeding of sweet potatoes.

## Data availability statement

The original contributions presented in this study are included in the article/**Supplementary Material**. Further inquiries can be directed to the corresponding author.

## Author contributions

Design of the study: ZS and LW; identification of *MYB* genes in the seven *Ipomoea* species: ZS and LW; molecular weight (MW) analysis: ZS, LW, and ZJ; isoelectric point (pI) analysis: ZS, LW, and MZ; subcellular localization analysis: ZS, ZJ, and YQ; motif analysis: ZS and YQ; gene structure analysis: ZS, LW, and ZJ; phylogenetic analysis: ZS, LW, and ZJ; chromosome location: ZS, KZ, and ZJ; duplication pattern analysis: ZS, LW, MZ, and ZJ; syntenic analysis: ZS, LW, MZ, and KZ; Ka/Ks analysis: ZS, KZ, MZ, and ZJ; expression profile analysis: ZS, KZ, MZ, and YQ; qRT-PCR analysis: ZS, LW, KZ, and YQ; manuscript preparation: ZS, LW, ZJ, MZ, KZ, and YQ. All authors contributed to the article and approved the submitted version.

## Supplementary material

Supplementary Figure 1 Sequence logos for the 20 conserved motifs of the MYB-related, R2R3-MYB, 3R-MYB, and 4R- and 5R-MYB proteins in the seven *Ipomoea* species, respectively.

Supplementary Figure 2 The phylogenetic tree of *Ipomoea* species and Arabidopsis 3R-MYB proteins. The proteins were grouped into 10 subgroups and each group has given a number (C1 to C10).

Supplementary Figure 3 The phylogenetic tree of *Ipomoea* species and Arabidopsis 4R- and 5R-MYB proteins.

Supplementary Figure 4 Motif and gene structure analyses of the *MYB* genes in the seven *Ipomoea* species. **(A)** Motif and gene structure analyses of the *MYB-related* genes in the seven *Ipomoea* species. **(B)** Motif and gene structure analyses of the *R2R3*-*MYB* genes in the seven *Ipomoea* species. **(C)** Motif and gene structure analyses of the *3R*-*MYB* genes in the seven *Ipomoea* species. **(D)** Motif and gene structure analyses of the *4R-* and *5R*-*MYB* genes in the seven *Ipomoea* species.

Supplementary Figure 5 The summary heatmap of *cis*-elements involved in plant growth and development and stress responses in *Ipomoea MYB* promoters.

Supplementary Figure 6
*Cis*-elements in the promoter region of *MYB* genes in *Ipomoea* species. **(A)** *Cis*-elements in *MYB-related* genes promoter; **(B)** *Cis*-elements in *R2R3-MYB* genes promoter; **(C)** *Cis*-elements in *3R-MYB* genes promoter; **(D)** *Cis*-elements in *4R-* and *5R-MYB* genes promoter.
