# Supplementary figures and images for: Comparative analysis of the MYB gene family in seven Ipomoea species
Source: Front Plant Sci. 2023 Mar 20;14:1155018. doi: 10.3389/fpls.2023.1155018 (PMC10067929; doi:10.3389/fpls.2023.1155018)

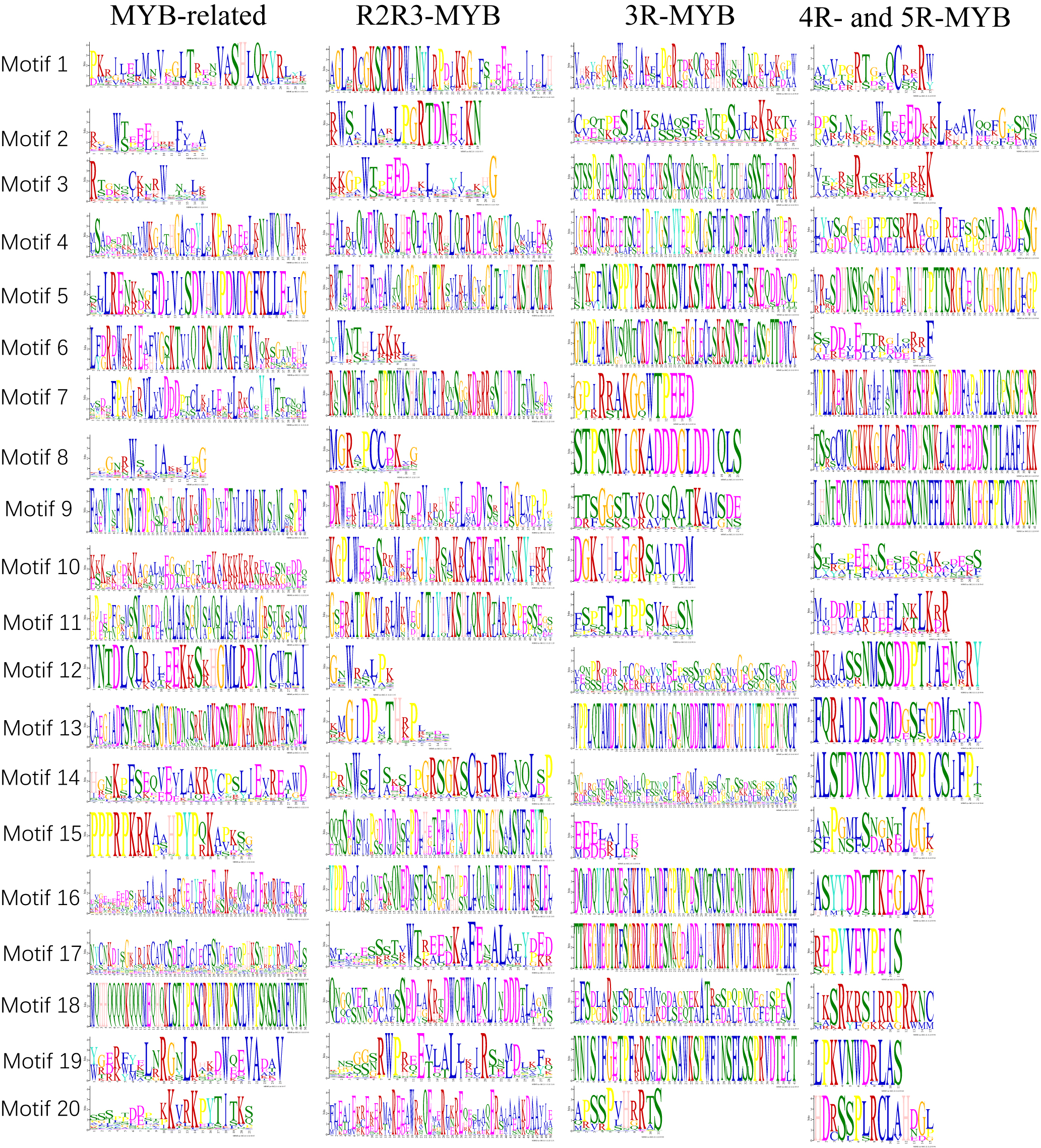

Supplement: Supplementary Figure 1 — Sequence logos for the 20 conserved motifs of the MYB-related, R2R3-MYB, 3R-MYB, and 4R- and 5R-MYB proteins in the seven Ipomoea species, respectively. [file Image_1.jpeg]

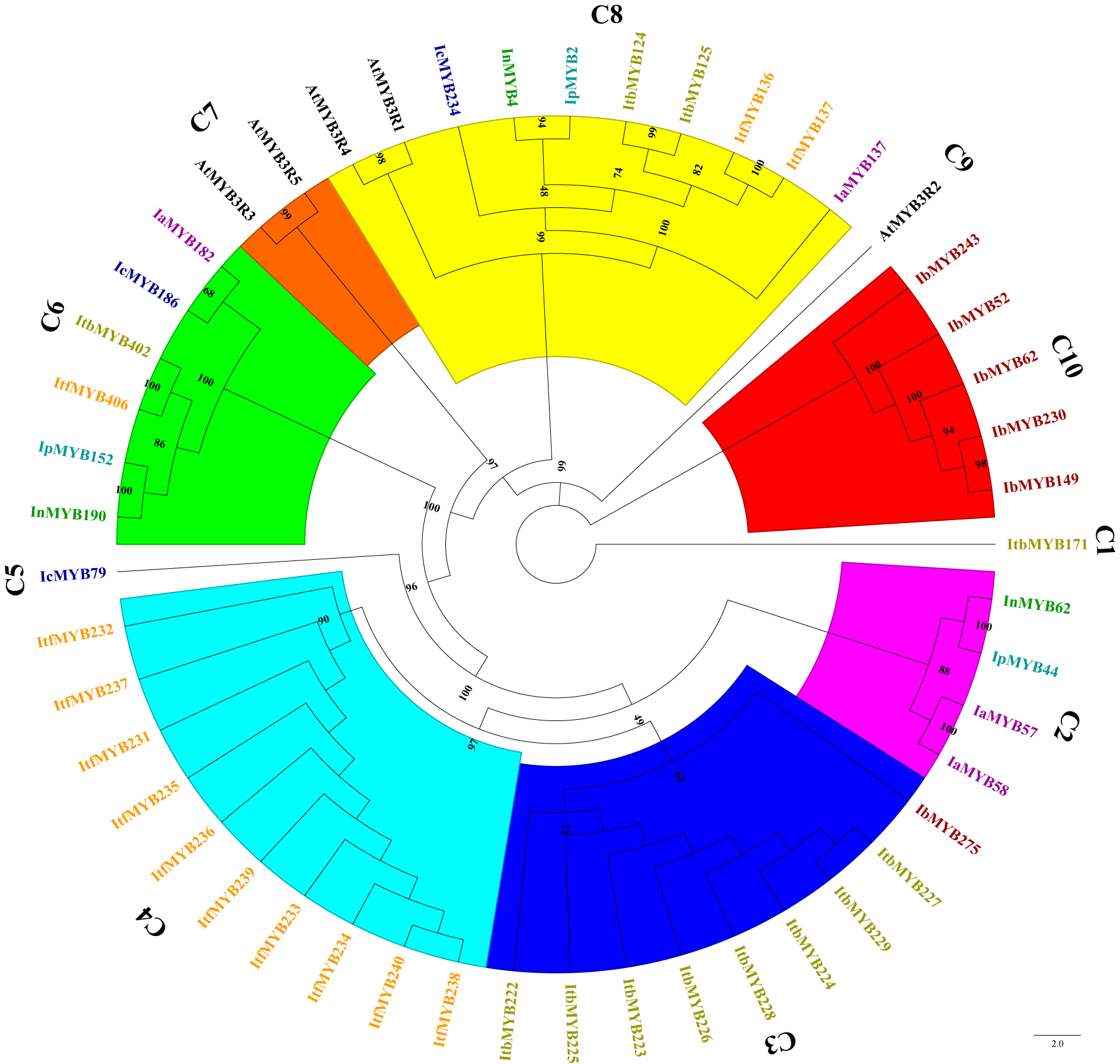

Supplement: Supplementary Figure 2 — The phylogenetic tree of Ipomoea species and Arabidopsis 3R-MYB proteins. The proteins were grouped into 10 subgroups and each group has given a number (C1 to C10). [file Image_2.jpeg]

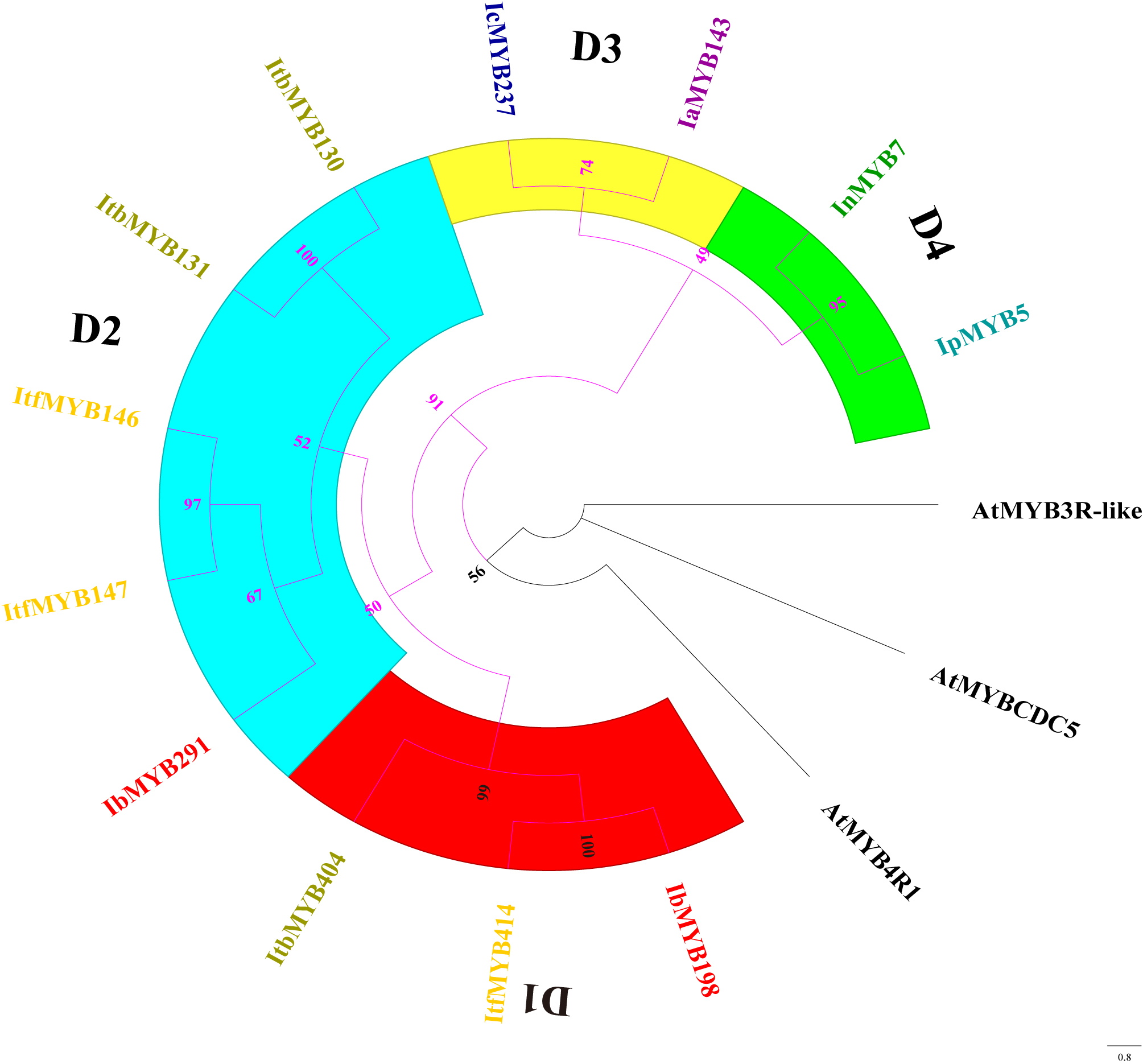

Supplement: Supplementary Figure 3 — The phylogenetic tree of Ipomoea species and Arabidopsis 4R- and 5R-MYB proteins. [file Image_3.jpeg]

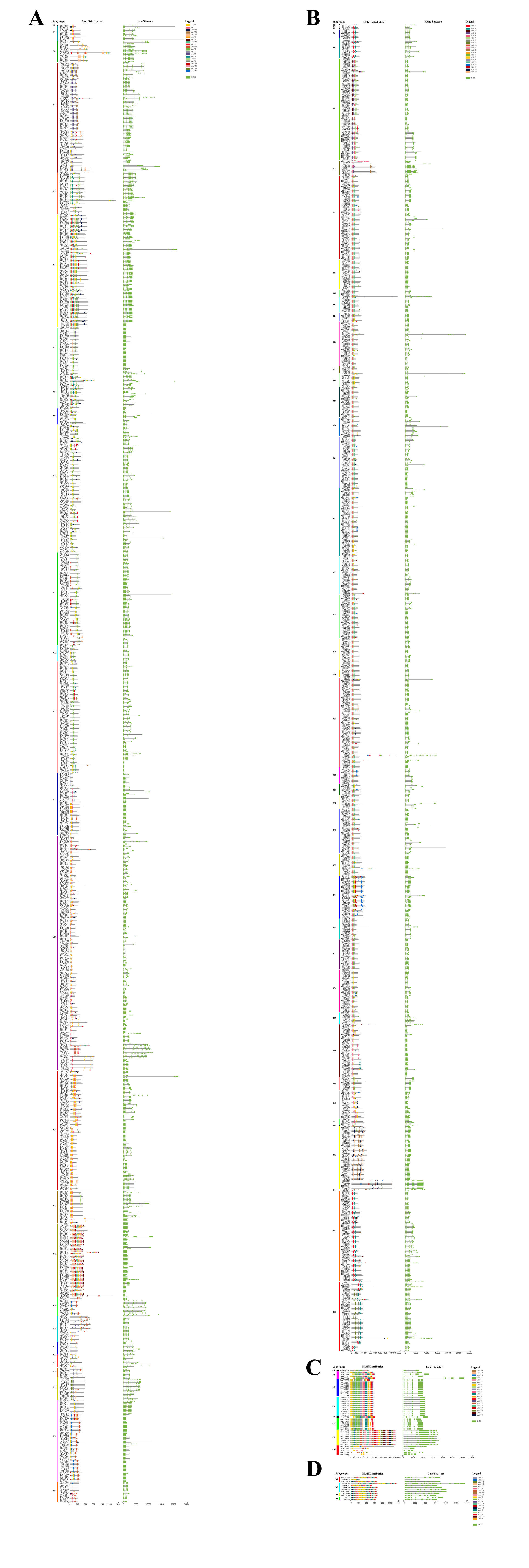

Supplement: Supplementary Figure 4 — Motif and gene structure analyses of the MYB genes in the seven Ipomoea species. (A) Motif and gene structure analyses of the MYB-related genes in the seven Ipomoea species. (B) Motif and gene structure analyses of the R2R3-MYB genes in the seven Ipomoea species. (C) Motif and gene structure analyses of the 3R-MYB genes in the seven Ipomoea species. (D) Motif and gene structure analyses of the 4R- and 5R-MYB genes in the seven Ipomoea species. [file Image_4.jpeg]

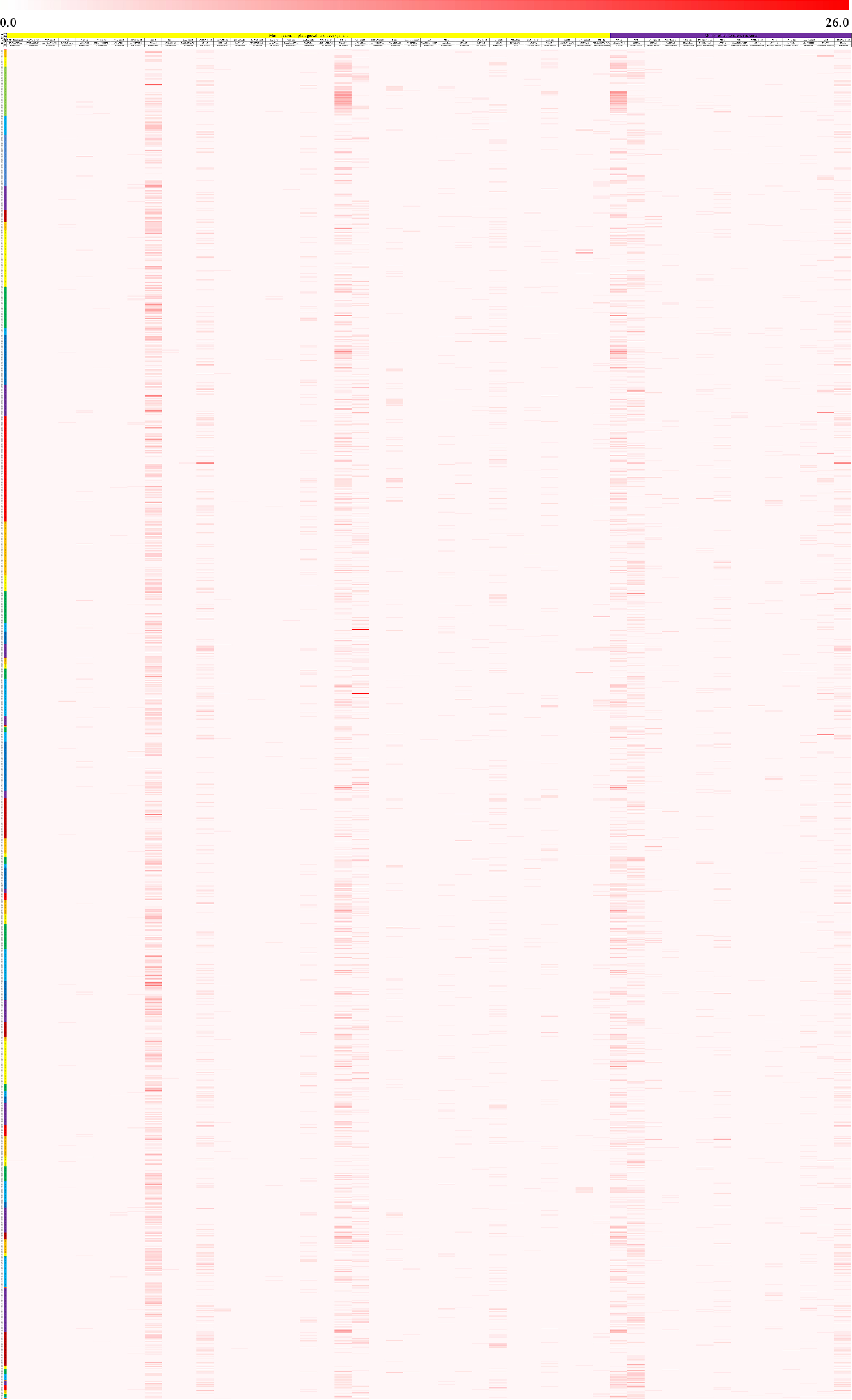

Supplement: Supplementary Figure 5 — The summary heatmap of cis-elements involved in plant growth and development and stress responses in Ipomoea MYB promoters. [file Image_5.jpeg]

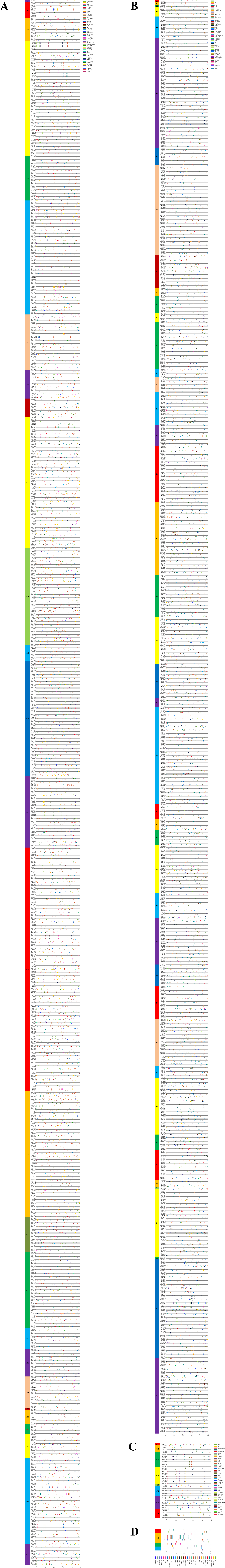

Supplement: Supplementary Figure 6 — Cis-elements in the promoter region of MYB genes in Ipomoea species. (A) Cis-elements in MYB-related genes promoter; (B) Cis-elements in R2R3-MYB genes promoter; (C) Cis-elements in 3R-MYB genes promoter; (D) Cis-elements in 4R- and 5R-MYB genes promoter. [file Image_6.jpeg]
